# Variation in the fibroblast growth factor 23 (*FGF23*) gene associates with serum FGF23 and bone strength in infants

**DOI:** 10.3389/fgene.2023.1192368

**Published:** 2023-05-22

**Authors:** Maria Enlund-Cerullo, Elisa Holmlund-Suila, Saara Valkama, Helena Hauta-alus, Jenni Rosendahl, Sture Andersson, Minna Pekkinen, Outi Mäkitie

**Affiliations:** ^1^ Children’s Hospital, Pediatric Research Center, University of Helsinki and Helsinki University Hospital, Helsinki, Finland; ^2^ Folkhälsan Research Center, Folkhälsan Institute of Genetics, Helsinki, Finland; ^3^ Research Program for Clinical and Molecular Metabolism, Faculty of Medicine, University of Helsinki, Helsinki, Finland; ^4^ Public Health Research, National Institute for Health and Welfare (THL), Helsinki, Finland; ^5^ PEDEGO Research Unit, MRC Oulu, Oulu University Hospital and University of Oulu, Oulu, Finland; ^6^ Department of Molecular Medicine and Surgery and Center for Molecular Medicine, Karolinska Institutet, and Clinical Genetics, Karolinska University Hospital, Stockholm, Sweden

**Keywords:** FGF23, genetic variation, vitamin D, bone strength, infants (0–24 months)

## Abstract

**Introduction:** The effects of genetic variation in fibroblast growth factor 23 (FGF23) are unclear. This study explores the associations of single-nucleotide polymorphisms (SNPs) of *FGF23* with phosphate and vitamin D metabolism and bone strength in early childhood.

**Methods:** The study is part of the vitamin D intervention in infant (VIDI) trial (2013–2016), in which healthy term infants born to mothers of Northern European origin received vitamin D_3_ supplementation of 10 or 30 μg/day from 2 weeks to 24 months of age (ClinicalTrials.gov NCT01723852). Intact and C-terminal FGF23 (cFGF23), 25-hydroxyvitamin D (25-OHD), parathyroid hormone, phosphate, and peripheral quantitative computed tomography (pQCT)-derived bone strength parameters were analyzed at 12 and 24 months. The study included 622 VIDI participants with genotyping data on *FGF23* SNPs rs7955866, rs11063112, and rs13312770.

**Results:** Rs7955866 minor allele homozygotes had lowest cFGF23 at both time-points (mixed model for repeated measurements, p_variant_ = 0.009). Minor alleles of rs11063112 were associated with a greater age-related decrease in phosphate concentration (p_interaction_ = 0.038) from 12 to 24 months. Heterozygotes of rs13312770 had the greatest total bone mineral content (total BMC), cross-sectional area (total CSA), and polar moment of inertia (PMI) at 24 months (ANOVA *p* = 0.005, 0.037, and 0.036, respectively). Rs13312770 minor alleles were associated with a greater increase of total BMC, but a smaller increase of total CSA and PMI, during follow-up (p_interaction_ <0.001, 0.043, and 0.012, respectively). Genotype of *FGF23* did not modify 25-OHD.

**Conclusion:** The study finds that genetic variation in *FGF23* modifies cFGF23, phosphate, and pQCT-derived bone strength parameters from 12 to 24 months of age. These findings potentially promote an understanding of the regulation of FGF23 and its role in bone metabolism and temporal changes thereof during early childhood.

## 1 Introduction

The fibroblast growth factor 23 (FGF23) hormone, produced mainly by bone osteocytes, participates in phosphate (Pi) and vitamin D metabolism (ADHR Consortium, 2000). FGF23 was initially identified in studies on autosomal dominant hypophosphatemic rickets (ADHR), in which mutations in the *FGF23* gene lead to excessive serum FGF23 concentration by preventing cleavage of the active intact FGF23 (iFGF23) into inactive N-terminal and C-terminal FGF23 (cFGF23). This results in higher iFGF23 concentrations and consequently in increased urinary phosphate loss (ADHR Consortium, 2000; White et al., 2001; Shimada et al., 2004; Takashi et al., 2018). Increased iFGF23 concentrations have, thereafter, been shown to be the underlying cause in several other genetic forms of hypophosphatemia (Shimada et al., 2001; Araya et al., 2005; Benet-Pagès et al., 2005; Martin et al., 2012; Gohil and Imel, 2019).

Phosphate is essential for bone mineralization and is mainly found in bone and intracellular compartments, with approximately 1% of total phosphate circulating in the blood stream. Circulating phosphate concentrations are tightly regulated by FGF23, 1,25 dihydroxyvitamin D (1,25-(OH)_2_D), and parathyroid hormone (PTH) (ADHR Consortium, 2000; Shimada et al., 2001; Erben and Andrukhova, 2017; Arnold et al., 2021). FGF23, in its intact form, directly reduces reabsorption of phosphate in the renal proximal tubules by suppressing the expression of sodium phosphate co-transporters, of which type IIa is encoded by the *SLC34A1* gene. FGF23 also affects phosphate concentrations indirectly by participating in the regulation of 1,25-(OH)_2_D concentration by, for example, increasing the expression of 25-hydroxyvitamin D-24-hydroxylase, a catabolic enzyme of vitamin D, encoded by the *CYP24A1* gene. Increased FGF23 leads to decreasing 1,25-(OH)_2_D concentrations and reduced intestinal phosphate uptake (Shimada, 2007; Martin et al., 2012; Quarles, 2012; Lederer, 2014; Takashi et al., 2018).

Circulating concentrations of phosphate and FGF23 show individual variation. Genome-wide association studies (GWAS) have identified several single-nucleotide polymorphisms (SNPs) associated with serum phosphate and FGF23 concentrations. SNPs near the genes *FGF23* and *SLC34A1* and *CASR*, the gene encoding the calcium sensing receptor, are associated with serum phosphate (Kestenbaum et al., 2010), while SNPs in genes involved in vitamin D metabolism, namely, *CYP24A1*, are associated with differences in circulating FGF23 concentrations (Robinson-Cohen et al., 2018).

The *FGF23* polymorphism rs7955866 (*SNP1*) is a missense variant located in the third exon of *FGF23* (c.716C>T, p.239T > M). Based on its location and the differing properties of the amino acid variants, rs7955866 is thought to significantly influence interactions of FGF23 with FGF receptors and Klotho, and thereby to have a role in phosphate homeostasis (Yamazaki et al., 2008; Rendina et al., 2012; Agoro et al., 2020). In adults with calcium nephrolithiasis, rs7955866 is associated with lower phosphate concentrations and increased urinary phosphate excretion (Rendina et al., 2012). *FGF23* variation has also been described to relate to cardiovascular outcomes, particularly in patients with chronic kidney disease. The 3′UTR variant rs11063112 (c.2185A>T) (*SNP2*) and intronic variant rs13312770 (c.211 + 3287A>G) (*SNP3*) have been associated with elevated risk of cardiovascular mortality in hemodialysis patients with chronic kidney disease and end-stage chronic kidney disease, respectively (Rothe et al., 2017; Schwantes-An et al., 2019).

We have previously reported that in infants aged 3–24 months, the concentrations and modifying factors of FGF23 depend on sex and age, iFGF23 being higher in girls and both iFGF23 and cFGF23 decreasing with age (Holmlund-Suila et al., 2016; Holmlund-Suila et al., 2017; Enlund-Cerullo et al., 2020). Furthermore, in a cohort of 183 school-aged children and adolescents, we have previously observed that common variants of *FGF23* (*SNP1*, *SNP2*, and rs3832879) show associations with PTH concentrations and urinary phosphate excretion, as well as with total hip bone mineral density (BMD) Z-score (Pekkinen et al., 2015), thereby linking *FGF23* variants to bone mass.

It remains unknown whether genetic variation in *FGF23* influences phosphate and vitamin D metabolism and bone strength during infancy. In this longitudinal cohort study, we examined associations of three *FGF23* polymorphisms (*SNP1–3*), with FGF23, phosphate, and vitamin D concentrations and with bone strength, determined by peripheral quantitative computed tomography (pQCT), from age 12–24 months in 622 healthy infants participating in a vitamin D intervention trial.

## 2 Methods

### 2.1 Participants

This study is part of the randomized and controlled vitamin D intervention in infant (VIDI) trial, which was carried out at the large, tertiary Kätilöopisto Maternity Hospital in Helsinki, Finland, from January 2013 to June 2016. We have previously reported the detailed protocol and exclusion criteria of VIDI (Helve et al., 2017; Rosendahl et al., 2018). In short, 975 healthy term infants, born to mothers of Northern European origin and with birthweight appropriate for gestational age, were randomized to receive vitamin D_3_ supplementation of either 10 μg (400 IU) (Group_10_) or 30 μg (1200 IU) (Group_30_) per day, from 2 weeks to 2 years of age.

### 2.2 Clinical and pQCT data collection

Data regarding the birth of the participating infants were obtained from medical records at recruitment. Cord blood samples were collected at birth and stored at −20°C for later extraction of genomic DNA. Study visits at 12 and 24 months included measurements of growth, venous blood samples, and bone strength, using peripheral quantitative computed tomography (pQCT) (Stratec XCT 2000 L Research+, Stratec Medizintechnik GmbH, Pforzheim, Germany) (Rosendahl et al., 2018; Valkama et al., 2020). Growth parameters were transformed into corresponding standard deviation scores (SDS) using Finnish pediatric growth references (Saari et al., 2011). FGF23, 25-hydroxyvitamin D (25-OHD), PTH, phosphate (Pi), ionized calcium (Ca ion), and iron concentrations were determined from the samples obtained at 12 and 24 months, stored at −80°C until analysis. The detailed study protocol and follow-up have previously been described (Helve et al., 2017; Rosendahl et al., 2018).

PQCT measurements were taken at 20% distal length of the left tibia. total BMC. Total vBMD, total CSA, and polar moment of inertia were determined. The image quality of the pQCT scans was graded based on the presence of motion artifacts (I = excellent, II = good, III = moderate, IV = sufficient, or V = poor), as previously reported (Rosendahl et al., 2018; Valkama et al., 2020). Scans with major motion artifacts or incorrect positioning of the examined limb were excluded.

Of 975 participants in VIDI, a total of 622 subjects with available genotyping data for one or more of the three studied SNPs of *FGF23* (rs7955866, rs11063112, and rs13312770) were included in the study. We have previously reported FGF23 concentrations and bone strength parameters in VIDI participants at 12 and 24 months, which were here included in analyses of association with *FGF23* genetic variation.

### 2.3 Genotype analysis

Genomic DNA from cord blood samples was extracted following the manufacturers’ instructions, using either automated Chemagen MSM1 extraction (PerkinElmer Inc., Chemagen Technologie GmbH, Baesweiler, Germany) or the Gentra Puregene kit (Qiagen GmgH, Hilden, Germany), at the laboratory of the Finnish Institute for Health and Welfare.

**FIGURE 1:**
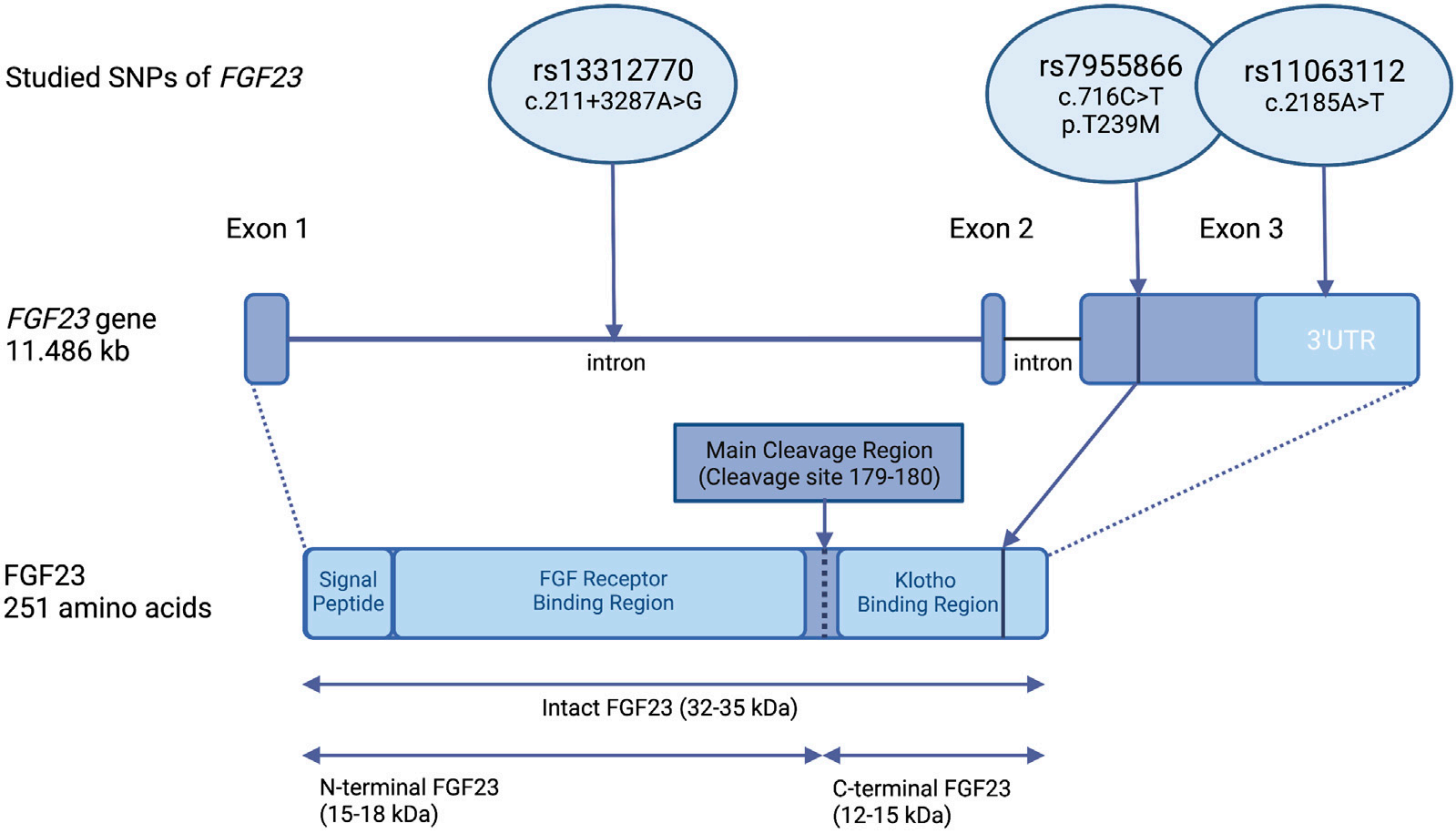
Location of the studied SNPs within the *FGF23* gene and protein. Organization of the *FGF23* gene and location of the studied variants; rs7955866 (*SNP1*), rs11063112 (*SNP2*), and rs13312770 (*SNP3*). Rs7955866 (c.716C>T), is a missense variant, located in the third exon of *FGF23*, which causes an amino acid change from threonine to methionine. The other two variants are located in non-translated regions of *FGF23*. Rs13312770 (c.211 + 3287A>G) is intronic, while rs110063112 (c.2185A>T) is located in the 3′- untranslated region (3′UTR) of *FGF23*. Based on the amino acid position, the variant rs7955866 is located in the Klotho binding region of FGF23 (position marked with a vertical line). Created with Biorender.com.

Based on previously demonstrated associations with phosphate and bone metabolism, *FGF23* SNPs rs7955866 (c.716C>T, p.239T > M) (*SNP1*) and rs11063112 (c.2185A>T) (*SNP2*) were chosen for the study (Rendina et al., 2012; Pekkinen et al., 2015). A third SNP, rs13312770 (c.211 + 3287A>G) (*SNP3*), was selected from the International HapMap project database (International HapMap Consortium, 2005) (http://www.ncbi.nlm.nih.gov/variation/tools/1000genomes/) using HaploView 4.2 software (Broad Institute, Cambridge, MA, United States; (http://www.broad.mit.edu/mpg/haploview) by tagging the SNP approach, using CEU population as a reference. The studied three tag SNPs (*SNP1–3*) also capture the variation in three other *FGF23* SNPs; rs13312789, rs13312786, and rs13312756. The localization of the selected SNPs in *FGF23* is shown in Figure 1.

The samples were genotyped by SNP-specific TaqMan Assays (Thermo-Fisher, Waltham, MA, United States) (TaqMan Assay IDs: C__25605491_20, C___2768724_10, and C__34067840_10 for *SNP1*, *SNP2*, and *SNP3*, respectively) and qPCR Bio-Rad CFX384 C1000 Touch™ Real-Time PCR Detection System (Bio-Rad, Hercules, CA, United States), in accordance with the manufacturer’s instructions. The amplification protocol used was 95°C for 3 min, followed by 39 cycles of 15 s at 92°C and 1 min at 60°C. End-point protocol analysis performed using CFX Manager 3.1 (Bio-Rad, Hercules, CA, United States) software was used to determine genotyping results. The obtained results were validated by simultaneous analysis of previously genotyped controls as well as randomly chosen internal duplicates and negative controls.

The obtained genotypes of the three studied SNPs were also combined into haplotypes using HaploView 4.2 software. In comparative analyses, identified haplotype homozygotes were used.

### 2.4 Biochemical analyses

Concentrations of biochemical parameters were determined from the samples obtained at 12 and 24 months of age.

FGF23, 25-OHD, and PTH concentrations were analyzed in the laboratories of the Pediatric Research Center at the University of Helsinki. Intact and C-terminal FGF23 concentrations were analyzed from venous blood sample plasma aliquots using commercially available enzyme-linked immunosorbent assay (ELISA) kits by KAINOS Laboratories (Tokyo, Japan) and Biomedica Medizinprodukte GmbH and Co. KG (Vienna, Austria) for determining iFGF23 and cFGF23 concentrations, respectively. Randomly chosen samples showed a mean CV% of 2% vs. 5% for iFGF23 and 7% vs. 3% for cFGF23 in duplicate analysis, at 12 vs. 24 months, respectively.

Concentrations of 25-OHD and PTH were analyzed by the fully automated IDS-iSYS immunoassay with chemiluminescence detection (Immunodiagnostic Systems Ltd., Bolton, United Kingdom), as previously reported (Rosendahl et al., 2018). The accuracy and quality of the methodology used were assessed by the vitamin D External Quality Assessment Scheme (DEQAS, Charing Cross Hospital, London, United Kingdom).

Analyses of Ca ion, Pi, and iron concentrations were performed by standard methods, at the accredited Central Laboratory of Helsinki University Hospital (HUSLAB).

### 2.5 Statistics

The results are presented as medians and interquartile ranges (IQRs) or means and 95% confidence intervals (95% CI). Normal distribution of variables was visually confirmed. Non-normally distributed variables were logarithmically converted (Ln) to enable parametric analyses. Differences between the sexes and intervention groups were examined by independent samples *t*-test or Pearson chi-squared test for categorical variables.

Differences between means of biochemical or bone parameters and genotypes of the studied SNPs were examined by analysis of variance (ANOVA) or Welch test of equality of means when variances were not equal. Multiple comparisons tests were performed using Bonferroni or Tamhane corrections. Mean allelic effects of the genotype were evaluated using multivariate linear regression. The temporal change in the studied biochemical and bone parameters and their associations with the genotype of the studied SNPs were examined using linear mixed models for repeated measurements. Covariates for linear regression and mixed-model analyses were selected based on previously reported associations with the studied parameters (Pekkinen et al., 2015; Holmlund-Suila et al., 2017; Rosendahl et al., 2018; Enlund-Cerullo et al., 2020; Valkama et al., 2020), and significant covariates were included in the models. For linear mixed-model analyses, covariates of continuous parameters included both 12- and 24-month measurements. All analyses were primarily performed including all participants, followed by the sex and intervention group. An overview of the performed analyses is presented in Supplementary Figure S1. IBM SPSS Statistics 24 (IBM, Armonk, NY, United States) software was used for statistical analyses. A *p*-value of <0.05 was considered statistically significant.

### 2.6 Study approval

The VIDI trial was approved by the Research Ethics Committee of the Hospital District of Helsinki and Uusimaa (107/13/03/03/2012) and carried out following the principles of the Helsinki Declaration. VIDI is registered in ClinicalTrials.gov (NCT01723852). Written informed consent was provided by the parents of participating infants at recruitment.

## 3 Results

### 3.1 Participant characteristics, biochemical findings, and bone parameters

This study included a total of 622 participants, with available genotyping data on three studied *FGF23* SNPs (64% of the original VIDI cohort; 53.7% girls). Key anthropometric, biochemical, and bone parameters of the participants at 12 and 24 months are reported by the sex and intervention group as given in Tables 1, 2, respectively. Boys were taller and heavier (*p* < 0.005 for both) and had lower iFGF23 (*p* < 0.001) and Ca ion (*p* < 0.002) than girls at both time points. PTH and iron concentrations were higher in girls at 24 months (*p* < 0.015). Due to the original recruitment schedule, most study visits were conducted during spring (Table 1). The levels of 25-OHD and PTH differed between intervention groups, with higher 25-OHD and correspondingly lower PTH concentrations in participants receiving high-dose vitamin D supplementation (Group_30_). Iron level was higher in Group_30_ at 12 months (*p* = 0.013) (Table 2).

**TABLE 1:** Participant characteristics at 12 and 24 months according to sex.

	12 months			24 months		
	Boys	Girls	*p*-value	Boys	Girls	*p*-value
Number of participants	288	334		288	334	
Length (cm)	76.0 (75.0:78.0)	74.0 (73.0; 76.0)	**<0.001**	88.0 (87.0; 90.0)	87.0 (85.0; 89.0)	**<0.001**
Weight (kg)	10.2 (9.4; 10.9)	9.3 (8.7; 10.0)	**<0.001**	12.8 (12.0; 13.5)	12.0 (11.3; 12.8)	**<0.001**
Length-adjusted weight SDS	0.08 (−0.59; 0.81)	−0.17 (−0.77; 0.62)	**0.004**	0.06 (−0.63; 0.58)	−0.10 (−0.84; 1.17)	0.321
Season at follow-up (N): winter/spring/summer/autumn^a^	58/114/61/55	61/140/74/59	0.866^b^	68/107/63/50	71/141/67/55	0.638
Onset of walking, age (months)	12 (11; 13)	12 (11; 13)	0.756	12 (11; 13)	12 (11; 13)	0.756
**Biochemical parameters** ^c^						
Intact FGF23 (pg/mL)	39.9 (34.2; 48.6)	45.0 (37.5; 52.0)	**<0.001** ^d^	39.4 (33.8; 45.8)	43.8 (36.3; 50.8)	**<0.001** ^d^
C-terminal FGF23 (pmol/L)	2.82 (2.14; 3.72)	2.91 (2.24; 3.78)	0.602^d^	1.97 (1.54; 2.75)	1.94 (1.46; 2.47)	0.174^d^
25-OHD (nmol/L)	93.8 (76.0; 115.2)	98.9 (78.1; 119.2)	0.164	99.1 (78.7; 120.1)	103.2 (83.4; 122.5)	0.123
Parathyroid hormone (pg/mL)^e^	23.3 (15.0; 31.9)	24.3 (15.9; 34.5)	0.436^d^	16.0 (10.7; 22.3)	17.2 (11.7; 23.9)	**0.014** ^d^
Phosphate (mmol/L)^e^	1.91 (1.82; 2.01)	1.91 (1.82; 2.01)	0.471	1.57 (1.47; 1.70)	1.59 (1.49; 1.72)	0.182
Ionized calcium (mmol/L)^e^	1.32 (1.30; 1.35)	1.34 (1.32; 1.36)	**<0.001**	1.31 (1.28; 1.33)	1.31 (1.29; 1.34)	**0.001**
Iron (μmol/L)^e^	10.5 (7.4; 13.8)	11.0 (7.6; 14.4)	0.305	12.3 (8.2; 16.0)	13.9 (10.0; 16.7)	**0.005**
**Bone parameters (N of scans)**	**229**	**269**		**286**	**331**	
Image quality: I/II/III/IV/V (N)^f^	24/59/65/41/40	53/66/84/39/37	0.268^b^	76/60/59/46/45	106/70/76/43/36	0.222^b^
Total BMC^g^ (mg/mm)	35.9 (31.2; 41.3)	33.3 (28.8; 37.1)	**<0.001**	55.7 (50.7; 60.6)	52.6 (47.3; 58.2)	**<0.001**
Total vBMD^g^ (mg/cm^3^)	297.0 (252.1; 347.7)	284.7 (237.6; 331.3)	0.064	368.2 (323.0; 428.3)	364.9 (319.2; 424.7)	0.340
Total CSA^g^ (mm^2^)	120.9 (103.0; 138.3)	114.6 (96.0; 137.8)	0.050	148.6 (132.5; 166.4)	141.3 (126.7; 161.3)	**0.004**
Polar moment of inertia	2,382 (1,728; 3,143)	2,162 (1,536; 3,094)	0.096	3,627 (2,916; 4,566)	3,315 (2,652; 4,269)	**0.004**

Data presented as medians (IQR) or N (%). *p*-values represent differences between sexes in independent samples *t*-test, unless otherwise specified. Significant p-values are highlighted in bold.

^a^ Winter = December, January, and February; spring, = March, April, and May; summer= June, July, and August; autumn = September, October, and November.

^b^ Pearson chi-square.

^c^ Number of participants if < 95% of total N in boys/girls, respectively: 12 months: iFGF23 (255/297), cFGF23 (251/284), 25-OHD (267/312), PTH (259/305), and Pi and Fe (228/263); 24 months: ionized calcium (268/304).

^d^ After logarithmic transformation.

^e^ Reference range: Parathyroid hormone 15–70 pg/mL, phosphate 1.3–2.2 mmol/L, ionized calcium 1.17–1.35 mmol/L, and iron 7–28 μmol/L.

^f^ Image quality grading in peripheral quantitative computed tomography (pQCT) scans: I, excellent; II, good; III, moderate; IV, sufficient; V = poor.

^g^ BMC, bone mineral content; vBMD, volumetric bone mineral density; CSA, total cross-sectional area of the bone.

**TABLE 2:** Participant characteristics at 12 and 24 months according to the intervention group.

	12 months			24 months		
	Group_10_	Group_30_	*p*-value	Group_10_	Group_30_	*p*-value
Number of participants (boys/girls)	306 (143/163)	316 (145/171)	0.832^a^	306 (143/163)	316 (145/171)	0.832^a^
Length (cm)	76.0 (74.0; 77.0)	75.0 (74.0; 77.0)	0.943	88.0 (86.0; 90.0)	88.0 (86.0; 90.0)	0.890
Weight (kg)	9.6 (8.9; 10.6)	9.6 (9.0; 10.4)	0.703	12.3 (11.4; 13.3)	12.4 (11.7; 13.2)	0.374
Length-adjusted weight SDS	−0.10 (−0.73; 0.70)	0.01 (−0.64; 0.71)	0.626	−0.12 (−0.95; 0.48)	0.04 (−0.62; 0.54)	0.142
Season at follow-up (N): winter/spring/summer/autumn^b^	56/132/60/58	63/122/75/56	0.504^a^	69/125/60/52	70/123/70/53	0.887^a^
Onset of walking, age (months)	12 (11; 13)	12 (11; 13)	0.159	12 (11; 13)	12 (11; 13)	0.159
**Biochemical parameters** *						
Intact FGF23 (pg/mL)	42.7 (35.8; 49.9)	43.2 (35.9; 51.6)	0.056^c^	40.8 (34.1; 47.2)	42.8 (36.5; 50.3)	0.150^c^
C-terminal FGF23 (pmol/L)	2.89 (2.24; 3.79)	2.86 (2.17; 3.67)	0.803^c^	1.95 (1.48; 2.63)	1.95 (1.50; 2.57)	0.637^c^
25-OHD (nmol/L)	80.3 (67.6; 97.2)	112.9 (96.4; 133.6)	**<0.001**	84.7 (72.8; 99.5)	117.0 (102.3; 135.2)	**<0.001**
Parathyroid hormone (pg/mL)^d^	25.4 (18.1; 34.7)	21.7 (14.0; 31.9)	**0.003** ^c^	17.9 (11.9; 31.2)	16.0 (11.1; 21.5)	**0.011** ^c^
Phosphate (mmol/L)^d^	1.91 (1.82; 2.01)	1.92 (1.81; 2.01)	0.591	1.59 (1.49; 1.69)	1.58 (1.47; 1.72)	0.908
Ionized calcium (mmol/L)^d^	1.33 (1.31; 1.35)	1.33 (1.31; 1.36)	0.100	1.31 (1.29; 1.33)	1.31 (1.29; 1.33)	0.764
Iron (μmol/L)^c^	10.1 (6.9; 13.2)	11.3 (7.9; 15.1)	**0.013**	12.9 (9.1; 16.3)	13.3 (9.4; 16.5)	0.241
**Biochemical parameters (N of scans)**	**247**	**251**		**301**	**316**	
Image quality: I/II/III/IV/V (N)^e^	34/54/70/42/47	33/71/79/38/30	0.147^a^	88/57/61/45/50	94/73/74/44/31	0.111^a^
Total BMC^f^ (mg/mm)	34.3 (29.7; 39.1)	34.0 (30.4; 38.9)	0.487	53.8 (48.3; 59.5)	53.9 (49.4; 58.9)	0.354
Total vBMD^f^ (mg/cm^3^)	287.5 (241.1; 346.0)	289.1 (247.2; 337.1)	0.988	364.4 (320.1; 425.7)	368.6 (322.9; 426.2)	0.780
Total CSA^f^ (mm^2^)	115.9 (99.1; 138.5)	117.4 (99.8; 137.4)	0.664	145.8 (129.3; 163.0)	145.4 (130.8; 164.9)	0.971
Polar moment of inertia	2181 (1641; 3152)	2239 (1639; 3075)	0.656	3468 (2727; 4355)	3470 (2802; 4452)	0.876

Data presented as medians (IQR) or N (%). *p*-values represent difference between groups in independent samples’ *t*-test, unless otherwise specified. Significant p-values are highlighted in bold.

^a^ Pearson chi-square.

^b^Winter = December, January, and February; spring = March, April, and May; summer = June, July, and August; autumn = September, October, and November.

^b^ After logarithmic transformation.

^c^ Reference range: Parathyroid hormone 15–70 pg/mL, phosphate 1.3–2.2 mmol/L, ionized calcium 1.17–1.35 mmol/L, and iron 7–28 μmol/L.

^d^ Image quality grading in peripheral quantitative computed tomography (pQCT) scans: I = excellent; II, good; III, moderate; IV, sufficient; V, poor.

^e^ BMC , bone mineral content; vBMD, volumetric bone mineral density; CSA, total cross-sectional area of the bone.

^f^ Number of participants if < 95% of total N in Group_10_/Group_30_, respectively: 12 months: iFGF23 (267/285), cFGF23 (259/276), 25-OHD (283/296), PTH (277/287), and Pi and Fe (241/250); 24 months: Ca (283/289).

Bone characteristics, determined by pQCT of the tibia, indicated that the total bone mineral content (total BMC) was lower in girls than in boys at both studied time points (*p* < 0.001 for both), and total cross-sectional area of the bone (total CSA) and polar moment of inertia were lower in girls at 24 months (*p* < 0.005) (Table 1). Bone parameters did not differ between intervention groups (Table 2).

### 3.2 Genotyping results and genotype distributions

Genomic DNA was extracted from cord blood samples, and genotypes were determined for three previously characterized variants of *FGF23*, using SNP-specific TaqMan Assays. Genotype call rates varied between 86% and 93%. The genotype for all three studied SNPs was determined for 526 (84.6%) participants. Obtained genotype distributions were in Hardy–Weinberg equilibrium, and in line with available previously reported genotype data (Zerbino et al., 2018; Lek et al., 2016; S isuproject, 2018). The studied three SNPs, rs7955866, rs11063112, and rs13312770 (S*NP1*, *SNP2*, and *SNP3*, respectively) were in linkage disequilibrium (D’ = 1, r2 = 0.02). Four different combinations of haplotype homozygotes for *SNP1–3* were observed; GTT (major allele homozygotes); GAT (major–minor–major homozygotes); GTC (major–major–minor homozygotes); and AAT (minor–minor–major allele homozygotes). Our study population did not include any subjects homozygous for all minor alleles. Genotype and haplotype distributions, presented in Table 3, did not differ between the sexes or intervention groups.

**TABLE 3:** Genotype and haplotype frequencies.

SNP	Variant	All	Group_10_	Group_30_	*p*-value^a^	Boys	Girls	*p*-value^a^
		N (%)	N (%)	N (%)		N (%)	N (%)	
**rs7955866**	GG	484 (81.8%)	242 (83.2%)	242 (80.4%)	0.449	232 (84.1%)	252 (79.7%)	0.266
(N = 592)	GA	101 (17.1%)	47 (16.2%)	54 (17.9%)		40 (14.5%)	61 (19.3%)	
	AA	7 (1.2%)	2 (0.7%)	5 (1.7%)		4 (1.4%)	3 (0.9%)	
**rs11063112**	TT	274 (46.6%)	142 (48.8%)	132 (44.4%)	0.508	129 (47.6%)	145 (45.7%)	0.694
(N = 588)	TA	260 (44.2%)	125 (43.0%)	135 (45.5%)		120 (44.3%)	140 (44.2%)	
	AA	54 (9.2%)	24 (8.2%)	30 (10.1%)		22 (8.1%)	32 (10.1%)	
**rs13312770**	TT	405 (73.0%)	204 (73.4%)	201 (72.6%)	0.976	183 (71.5%)	222 (74.2%)	0.413
(N = 555)	TC	138 (24.9%)	68 (24.5%)	70 (25.3%)		69 (27.0%)	69 (23.1%)	
	CC	12 (2.2%)	6 (2.2%)	6 (2.2%)		4 (1.6%)	8 (2.7%)	
**Haplotype**	GTT	149 (78.8%)	84 (81.6%)	65 (75.6%)	0.430	72 (82.8%)	77 (75.5%)	0.447
(N = 189)	GAT	23 (12.2%)	12 (11.7%)	11 (12.8%)		8 (9.2%)	15 (14.7%)	
	GTC	12 (6.3%)	6 (5.8%)	6 (4.7%)		4 (4.6%)	8 (7.8%)	
	AAT	5 (2.6%)	1 (1.0%)	4 (4.7%)		3 (3.4%)	2 (2%)	

^a^ Pearson chi-square.

### 3.3 Associations of genotype and biochemical and bone parameters

**FIGURE 2:**
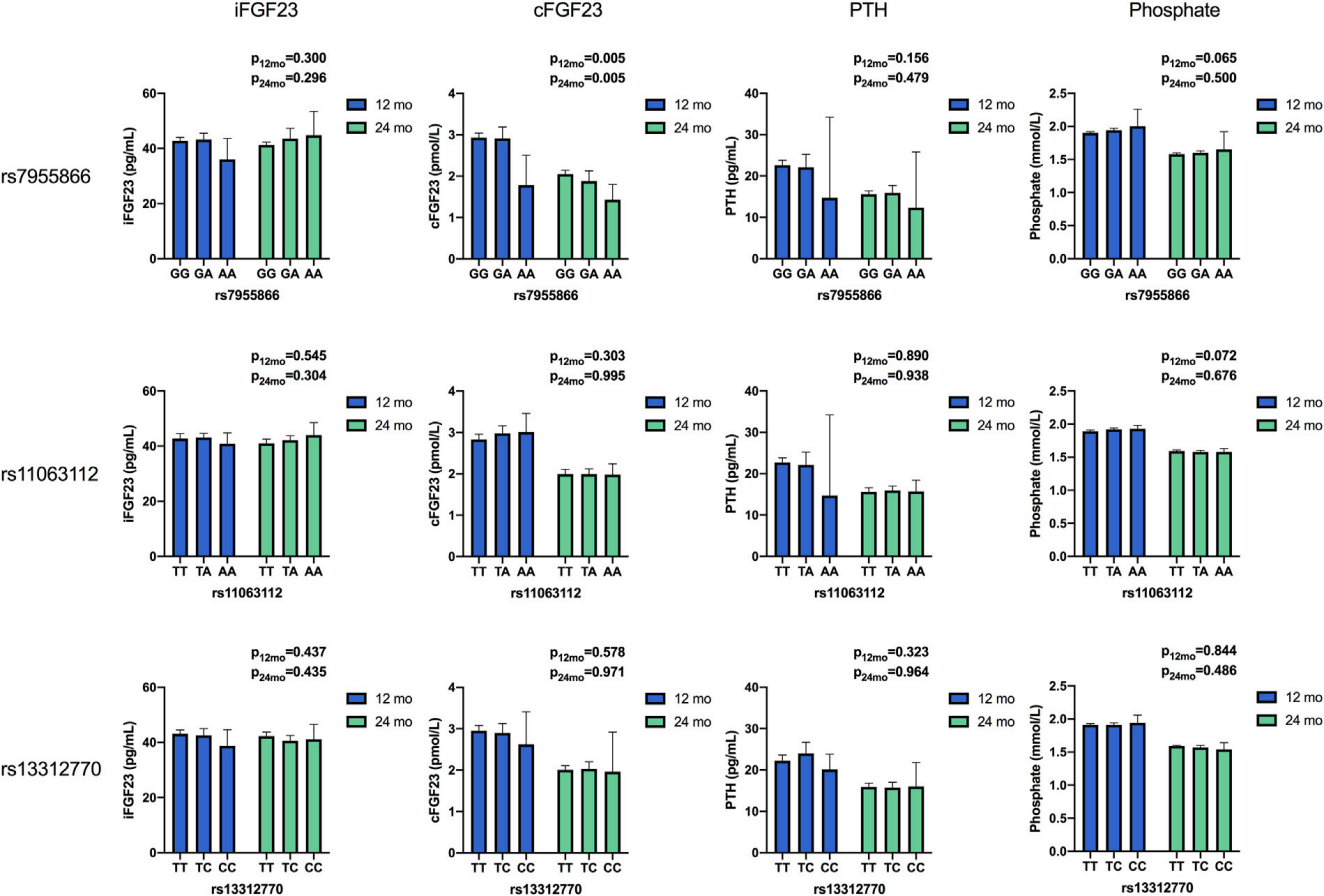
Unadjusted mean concentrations of biochemical parameters by the genotype of studied SNPs at 12 and 24 months. Results for differences between variants in ANOVA. (iFGF23, intact fibroblast growth factor 23; cFGF23, C-terminal fibroblast growth Factor 23; PTH, parathyroid hormone).

**FIGURE 3:**
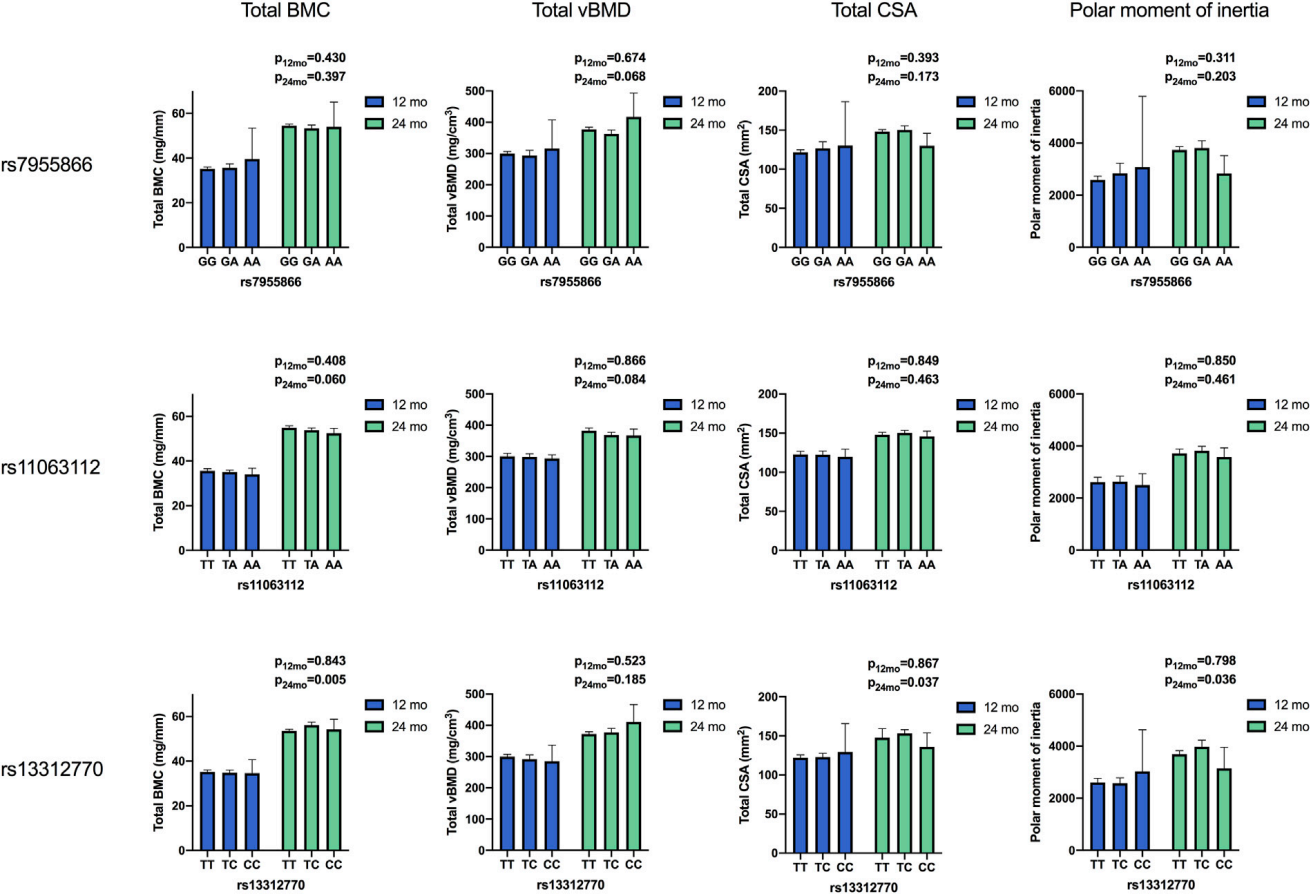
Means of bone parameters by the genotype of studied SNPs at 12 and 24 months. Results for differences between variants in ANOVA. (BMC, bone mineral content; vBMD, volumetric bone mineral density; CSA, cross-sectional area of the bone).

Mean unadjusted concentrations by genotype and haplotype and results for analyses of differences between variants are presented in Figures 2, 3 and Tables 4, 5. The adjusted mean allelic effects of the studied genotypes on biochemical and bone parameters at 12 and 24 months are presented in Table 6.

**TABLE 4:** Biochemical variables by the genotype at 12 and 24 months and difference between variants in ANOVA.

SNP	Variant (N)	iFGF23 (pg/mL)^a^	*p*-value	cFGF23 (pmol/L)^a^	*p*-value	PTH (pg/mL)^a^	*p*-value	Phosphate (mmol/L)	*p*-value
**12 months**									
**rs7955866**	GG (223)	42.7 (41.5; 44.0)	0.300	2.93 (2.82; 3.04)	**0.005** ^b^ ^,^ ^c^	22.6 (21.4; 23.8)	0.156^d^	1.90 (1.88; 1.92)	0.065^e^
	GA (54)	43.2 (41.0; 45.5)		2.91 (2.65; 3.19)		22.1 (19.4; 25.2)		1.94 (1.91; 1.97)	
	AA (7)	36.0 (30.5; 43.6)		1.78 (1.26; 2.51)		14.7 (6.3; 34.2)		2.00 (1.74; 2.26)	
**rs11063112**	TT (246)	42.7 (41.0; 44.5)	0.545^f^	2.83 (2.70; 2.96)	0.303^g^	22.7 (21.2; 24.4)	0.890	1.89 (1.87; 1.91)	0.072^h^
	TA (226)	43.1 (41.6; 44.6)		2.98 (2.82; 3.16)		22.3 (20.7; 24.0)		1.92 (1.90; 1.94)	
	AA (48)	40.9 (37.3; 44.8)		3.01 (2.61; 3.46)		21.9 (18.1; 26.5)		1.93 (1.88; 1.98)	
**rs13312770**	TT (358)	43.1 (41.8; 44.5)	0.437^i^	2.95 (2.83; 3.08)	0.578	22.2 (20.9; 23.6)	0.323	1.91 (1.90; 1.93)	0.844
	TC (122)	42.5 (40.1; 45.0)		2.90 (2.68; 3.13)		24.0 (21.6; 26.7)		1.91 (1.88; 1.94)	
	CC (12)	38.7 (33.5; 44.6)		2.62 (2.02; 3.41)		20.1 (13.5; 23.8)		1.94 (1.82; 2.06)	
**Haplotype**	GTT (117)	43.0 (40.7; 45.5)	0.435	2.87 (2.70; 3.05)	**0.034** ^j^	23.3 (21.2; 25.5)	0.295^k^	1.89 (1.86; 1.92)	0.269^L^
	GAT (19)	41.2 (34.0; 49.9)		3.21 (2.61; 3.94)		27.4 (22.2; 33.8)		1.87 (1.79; 1.96)	
	GTC (10)	38.7 (33.5; 44.6)		2.62 (2.02; 3.41)		20.1 (13.5; 29.8)		1.94 (1.82; 2.06)	
	AAT (4)	35.7 (27.4; 46.6)		1.89 (1.11; 3.02)		17.9 (5.8; 55.1)		2.02 (1.64; 2.40)	
**24** **months**									
**rs7955866**	GG (484)	41.2 (40.1; 42.3)	0.296^g^	2.05 (1.97; 2.14)	**0.005** ^g^ ^,^ ^m^	15.6 (14.9; 16.4)	0.479^n^	1.58 (1.57; 1.60)	0.500
	GA (101)	43.5 (40.0; 47.3)		1.88 (1.66; 2.13)		15.9 (14.3; 17.7)		1.60 (1.56; 1.63)	
	AA (7)	44.8 (37.6; 53.4)		1.43 (1.14; 1.80)		12.3 (5.9; 25.8)		1.65 (1.38; 1.92)	
**rs11063112**	TT (274)	41.0 (39.5; 42.5)	0.304	1.99 (1.8; 2.11)	0.995	15.6 (14.7; 16.6)	0.938	1.59 (1.57; 1.61)	0.676
	TA (260)	42.1 (40.4; 43.8)		1.99 (1.87; 2.12)		15.9 (14.9; 17.0)		1.58 (1.56; 1.60)	
	AA (54)	44.0 (39.9; 48.5)		1.98 (1.74; 2.24)		15.7 (13.3; 18.4)		1.58 (1.53; 1.63)	
**rs13312770**	TT (405)	42.3 (40.9; 43.8)	0.435	2.01 (1.91:2.11)	0.971	15.9 (15.1; 16.8)	0.964	1.59 (1.57; 1.60)	0.486
	TC (138)	40.6 (38.8; 42.5)		2.03 (1.87; 2.20)		15.7 (14.5; 17.0)		1.57 (1.55; 1.60)	
	CC (12)	41.1 (36.3; 46.5)		1.96 (1.32; 2.92)		16.0 (11.8; 21.8)		1.54 (1.44; 1.64)	
**Haplotype**	GTT (149)	41.2 (39.0; 43.6)	0.966	1.95 (1.80; 2.12)	0.382	16.4 (15.1; 17.8)	0.780	1.60 (1.57; 1.63)	0.308
	GAT (23)	41.1 (36.8; 45.8)		2.24 (1.84; 2.72)		17.9 (14.6; 21.9)		1.54 (1.48; 1.61)	
	GTC (12)	41.1 (36.3; 46.5)		1.96 (1.32; 2.92)		16.0 (11.8; 21.8)		1.54 (1.44; 1.64)	
	AAT (5)	44.5 (35.1; 56.3)		1.50 (1.12; 2.00)		14.1 (5.2; 38.2)		1.61 (1.13; 2.08)	

Concentrations reported as means and 95% CI, *p*-values reflect differences between genotypes in ANOVA (unless otherwise specified).

^a^ Back-transformed from logarithmic transformation.

^b^ Significant differences in multiple comparisons: AA vs. GG and AA vs. GA (Bonferroni *p* = 0.003 and 0.006, respectively).

^c^ cFGF23: In boys: *p* = 0.025 (Bonferroni AA vs. GG *p* = 0.038).

In Group_30_: *p* = 0.022 (AA vs. GG and GA, Bonferroni *p* = 0.030 and 0.017, respectively, mean difference: 1.60 and 1.70, respectively).

^d^ PTH: In Group_10_: *p* < 0.001 (AA vs. GA and GG, Bonferroni *p* < 0.001 for both, mean difference: 5.8 and 5.5, respectively).

^e^ Pi: In Group_10_: *p* = 0.012, multiple comparisons not performed due to low N.

^f^ iFGF23: In boys: *p* = 0.041 (TT vs. AA Bonferroni *p* = 0.035; mean difference: 1.2).

^g^ Welch test of equality of means.

^h^ Pi: In Group_10_: *p* = 0.003 (TA vs. TT, Bonferroni *p* = 0.005, mean difference: 0.06).

^i^ iFGF23: In girls: *p* = 0.019; NS in multiple comparisons.

^j^ Significant differences in multiple comparisons: GAT vs. AAT (Bonferroni *p* = 0.029, mean difference 1.69), In Group_10_: *p* = 0.047.

^k^ PTH: In Group_10_: *p* = 0.008, multiple comparisons not performed due to low N.

^l^ Pi: In boys: *p* = 0.034 (GTC vs. GAT Bonferroni *p* = 0.032, mean difference: 0.26).

^m^ Significant differences in multiple comparisons: GG vs. AA (Tamhane *p* = 0.023).

In boys: ANOVA: *p* = 0.043, NS, in multiple comparisons (Welch *p* < 0.001, Tamhane: GG vs. AA *p* < 0.001, mean difference: 1.32).

In Group_30_: Welch *p* = 0.021, Tamhane NS.

^n^ PTH: In boys: ANOVA = 0.027, (GG vs. AA, Bonferroni *p* = 0.025, mean difference 2.1).

In Group_10_: *p* = 0.003 (AA vs. GG and GA, Bonferroni *p* = 0.002 for both, mean differences: 3.8 and 3.9, respectively).

Number of participants if <95% of total N: phosphate at 12 months: rs7955966 (GG/GA/AA): 383/82/5, rs11063112 (TT/TA/AA): 214/208/45, rs11312770 (TT/TC/CC): 324/104/10.

iFGF23, intact fibroblast growth factor 23; cFGF23, C-terminal fibroblast growth factor 23; PTH, parathyroid hormone.

**TABLE 5:** Bone parameters by the genotype at 12 and 24 months and difference between variants in ANOVA.

SNP	Variant (N)	Total BMC (mg/mm)	*p*-value	Total vBMD (mg/cm^3^)	*p*-value	Total CSA (mm^2^)	*p*-value	Polar moment of inertia	*p*-value
**12 months**									
**rs7955866**	GG (386)	35.2 (34.4; 36.0)	0.430^a^	299.5 (292.3; 306.6)	0.674	121.7 (118.6; 124.9)	0.393^b^	2582 (2438; 2727)	0.311^c^
	GA (79)	35.6 (33.7; 37.4)		293.2 (276.4; 310.0)		126.8 (118.6; 135.1)		2838 (2451; 3226)	
	AA (5)	39.6 (25.9; 53.4)		316.0 (224.7; 407.3)		130.3 (74.2; 186.4)		3077 (362; 5793)	
**rs11063112**	TT (216)	35.6 (34.5.36.6)	0.408^d^	300.0 (290.2; 309.8)	0.866	122.7 (118.6; 126.8)	0.849	2612 (2428; 2795)	0.850
	TA (210)	35.0 (34.1; 35.9)		298.7 (289.1; 308.4)		122.4 (117.8; 127.0)		2632 (2422; 2841)	
	AA (44)	34.0 (31.1; 36.8)		293.6 (292.3; 305.4)		119.7 (110.0; 129.4)		2495 (2051; 2938)	
**rs13312770**	TT (327)	35.2 (34.4; 36.1)	0.843	299.6 (292.0; 307.3)	0.523	121.9 (118.4; 125.5)	0.867^e^	2601 (2439; 2762)	0.798^e^
	TC (107)	34.8 (33.5; 36.0)		291.7 (278.2; 305.3)		122.9 (118.0; 127.7)		2574 (2365; 2783)	
	CC (9)	34.6 (28.5; 40.7)		285.2 (234.0; 336.4)		129.3 (92.9; 165.6)		3043 (1460; 4627)	
**Haplotype**	GTT (119)	35.7 (34.1; 37.3)	0.667	297.6 (283.7; 311.5)	0.807	123.9 (118.3; 129.6)	0.694	2663 (2414; 2911)	0.577
	GAT (20)	33.1 (29.6; 36.7)		288.6 (259.9; 317.3)		118.0 (104.5; 131.4)		2388 (1869; 2907)	
	GTC (9)	34.6 (28.5; 40.7)		285.2 (234.0; 336.4)		129.3 (92.9; 165.6)		3043 (1460; 4627)	
	AAT (4)	34.9 (29.1; 40.7)		323.3 (191.7; 455.0)		111.8 (78.8; 144.7)		2147 (902; 3393)	
**24** **months**									
**rs7955866**	GG (482)	54.5 (53.8; 55.2)	0.397	377.2 (370.3; 384.0)	0.068^f^	148.3 (145.9; 150.8)	0.173	3738 (3612; 3864)	0.203
	GA (99)	53.3 (51.8; 54.8)		362.5 (349.6; 375.4)		150.2 (144.8; 155.6)		3810 (3533; 4087)	
	AA (7)	54.0 (42.9; 65.1)		417.3 (341.7; 493.0)		130.1 (114.3; 145.9)		2831 (2147; 3515)	
**rs11063112**	TT (274)	54.9 (54.0; 55.8)	0.060^g^	381.8 (372.7; 390.9)	0.084^h^	147.8 (144.5; 151.1)	0.463	3713 (3544; 3881)	0.461
	TA (257)	53.8 (52.9; 54.8)		368.0 (358.9; 377.2)		150.1 (146.7; 153.5)		3815 (3642; 3989)	
	AA (53)	52.4 (50.1; 54.6)		367.0 (346.4; 387.7)		145.7 (138.9; 152.5)		3577 (3228; 3927)	
**rs13312770**	TT (400)	53.5 (52.8; 54.3)	**0.005** ^i^	372.1 (364.7; 379.5)	0.185^j^	147.5 (144.9; 159.2)	**0.037** ^k^ ^,^ ^l^	3688 (3553; 3823)	**0.036** ^k^ ^,^ ^m^
	TC (138)	56.1 (54.7; 57.5)		377.3 (364.3; 390.4)		153.0 (148.0; 157.9)		3977 (3723; 4229)	
	CC (12)	54.2 (49.6; 58.8)		411.2 (356.0; 466.4)		135.9 (119.1; 153.7)		3142 (2332; 2953)	
**Haplotype**	GTT (149)	54.2 (52.9; 55.4)	0.669	378.0 (365.8; 390.2)	0.540	146.9 (1142.6; 151.3)	0.329	3666 (3442; 3891)	0.361
	GAT (23)	53.6 (49.4; 57.8)		374.4 (335.8; 412.9)		146.8 (135.4; 158.1)		3643 (3063; 4222)	
	GTC (12)	54.2 (49.6; 58.8)		411.2 (356.0; 466.4)		135.9 (118.1; 153.7)		3142 (2332; 3953)	
	AAT (5)	49.8 (43.8; 55.7)		384.9 (303.6; 466.3)		131.0 (110.6; 151.4)		2851 (1952; 3749)	

Concentrations reported as means and 95% CI; *p*-values reflect differences between the genotype in ANOVA (unless otherwise specified).

^a^ Total BMC; Group_10_: *p* = 0.006, multiple comparisons not performed due to low N.

^b^ Total CSA; Group_10_: *p* = 0.012, multiple comparisons not performed due to low N.

^c^ Polar moment of inertia; Group_10_: *p* = 0.006, multiple comparisons not performed due to low N.

^d^ Total BMC: In boys: Welch *p* = 0.005, significant differences in multiple comparisons (Tamhane) TT vs. AA (*p* = 0.035, mean difference: 5.0). In Group_10_: *p* = 0.031, significant differences (Bonferroni) TT vs. AA (*p* = 0.039, mean difference: 4.3).

^e^ Welch test of equality of means.

^f^ Total vBMD: In girls: Welch *p* < 0.001, significant differences in multiple comparisons (Tamhane) AA vs. GG and GA (*p* < 0.001 for both, mean difference: 75.9 and 88.9, respectively).

^g^ Total BMC: In boys: *p* = 0.003, significant differences in multiple comparisons (Bonferroni) TT vs. TA (*p* = 0.005, mean difference: 3.8).

^h^ Total vBMD. In boys: *p* = 0.032, significant differences in multiple comparisons (Bonferroni) TT vs. TA (*p* = 0.004, mean difference: 24.4).

^i^ Significant differences in multiple comparisons (Bonferroni) TT vs. TC (*p* = 0.004). In boys: *p* = 0.037, significant differences (Bonferroni) TT vs. TC (*p* = 0.026, mean difference: 2.9), In Group_10_: *p* = 0.024, significant differences (Bonferroni) TT vs. TC (*p* = 0.021, mean difference: 3.1).

^j^ In Group_10_ *p* = 0.045, Bonferroni NS.

^k^ Multiple comparisons NS.

^l^ Group_30_: *p* = 0.005, significant differences (Bonferroni) TT vs. TC (*p* = 0.005, mean difference: 11.3).

^m^ Group_30_: *p* = 0.005, significant differences (Bonferroni) TT vs. TC (*p* = 0.005, mean difference: 576).

BMC, bone mineral content; vBMD, volumetric bone mineral density; CSA, cross-sectional area of the bone.

**TABLE 6:** Adjusted mean allelic effects of studied variants on biochemical and bone parameters at 12 and 24 months.

	rs7955866 (G>A)		rs11063112 (T>A)		rs13312770 (T>C)	
	B (95% CI)	P_Adj_	B (95% CI)	P_Adj_	B (95% CI)	P_Adj_
**iFGF23 (pg/mL) ^a^** 12 months	−1.86 (−6.06 to 2.33)	0.383	−1.14 (−3.90 to 1.62)	0.416	−0.34 (−4.10 to 3.42)	0.858 ^**b**^
24 months	5.56 (0.12–11.01)	**0.045 ^b^**	2.11 (−1.43–5.65)	0.242	−3.47 (−8.39 to 1.45)	0.167
**cFGF23 (pmol/L) ^c^** 12 months	−0.97 (−0.42 to 0.23)	0.556	0.23 (0.02–0.44)	**0.030 ^d^**	−0.06 (−0.35 to 0.24)	0.709
24 months	0.16 (−0.36–0.67)	0.549 ^**e**^	0.00 (−0.33–0.34)	0.992	−0.12 (−0.58 to 0.34)	0.600
**PTH (pg/mL) ^f^** 12 months	−1.02 (−3.70 to 1.65)	0.452	−0.01 (−1.78 to 1.75)	0.987	1.22 (−1.11–3.56)	0.304
24 months	−0.51 (−2.26 to 1.25)	0.573	0.37 (−0.78–1.53)	0.525	−0.25 (−1.74 to 1.23)	0.738
**Phosphate (mmol/L) ^g^** 12 months	0.04 (0.00–0.07)	**0.027 ^h^**	0.03 (0.01–0.05)	**0.017 ^h^**	0.00 (−0.02–0.03)	0.793
24 months	0.02 (−0.02–0.05)	0.298	−0.01 (−0.03 to 0.01)	0.446	−0.02 (−0.04 to 0.01)	0.267
**Total BMC (mg/mm) ^i^** 12 months	1.0 (−0.6–2.6)	0.202	−0.4 (−1.4 to 0.6)	0.462	−0.9 (−2.3 to 0.4)	0.181
24 months	−0.5 (−1.9 to 0.9)	0.490	−0.8 (−1.7 to 0.1)	0.076 ^**j**^	1.4 (0.2–2.6)	**0.028**
**Total vBMD (mg/cm** ^**3**^ **) ^i^** 12 months	−2.2 (−17.8; 13.4)	0.784	−2.5 (−12.7 to 7.7)	0.632	−8.1 (−21.4 to 5.3)	0.237
24 months	−5.7 (−20.1 to 8.7)	0.438	−9.5 (−19.0 to 0.0)	0.050	8.1 (−4.6–20.9)	0.210 **^k^**
**Total CSA (mm** ^**2**^ **) ^i^** 12 months	5.4 (−1.5–12.3)	0.125 ^**L**^	0.1 (−4.3–4.5)	0.971	0.2 (−5.6–6.0)	0.943
24 months	0.3 (−4.9–5.4)	0.922	1.2 (−2.2–4.5)	0.500	1.3 (−3.2–5.8)	0.584 **^L^**
**Polar moment of inertia ^i^** 12 months	273 (−47–592)	0.094 **^m^**	22 (−177–221)	0.828	−20 (−281 to 241)	0.881
24 months	−1 (−263 to 261)	0.991	44 (−129–216)	0.617	83 (−147–313)	0.478 **^m^**

Adjusted mean allelic effects for the studied SNPs on biochemical and bone parameters. Results for multivariate linear regression analyses. Values are reported as B coefficients and 95% confidence intervals (95% CI). Significant p-values are highlighted in bold.

^a^ Covariates: sex, iron, ionized calcium, and 25-hydroxyvitamin D at 12 and 24 months.

^b^ Significant adjusted mean allelic effects on iFGF23 in subset analyses: At 12 months; rs13312770: In girls: B= 4.61 (95% CI –7.90 to −1.32), *p* = 0.006. At 24 months; rs7955866: In Group_10_: B = 12.95 (95% CI, 2.00–23.90), *p* = 0.021: In girls: B = 11.72 (95% CI, 2.87–20.57), *p* = 0.010.

^c^ Covariates: Iron at 12 and 24 months, respectively.

^d^ Significant adjusted mean allelic effects on cFGF23 in subset analyses: Rs11063112 in girls at 12 months: B 0.30 (95% CI, 0.20–0.58), *p* = 0.036. In secondary analyses using logarithmic conversions due to non-optimally normally distributed residuals: Rs11063112 in all at 12 months *p* = 0.118, in girls B 1.06 (95% CI, 1.01–1.12), *p* = 0.030.

^e^ Significant adjusted allelic effects on cFGF23 in secondary analyses using logarithmic conversions due to non-normally distributed residuals: Rs7955866 at 24 months in all participants, *p* = 0.024.

^f^ Covariates: sex, ionized calcium, and 25-hydroxyvitamin D at 12 and 24 months.

^g^ Covariates: sex, iron, length-adjusted weight SDS, and parathyroid hormone (after logarithmic conversion) at 12 and 24 months.

^h^ Significant adjusted mean allelic effects on phosphate in subset analyses: At 12 months: rs7955866: Group_10_: B 0.06 (95% CI, 0.01–0.11) *p* = 0.011, rs11063112: In Group_10_: B 0.05 (95% CI, 0.02–0.07), *p* = 0.001. In girls: B 0.03 (95% CI, 0.00–0.06), *p* = 0.043.

^i^ Covariates: image quality, sex, and length-adjusted weight SDS at 12 and 24 months.

^j^ Significant adjusted mean allelic effects on total BMC in subset analyses at 24 months: rs11063112: In boys: B: 1.9 (95% CI -3.3 to −0.5), *p* = 0.008.

^k^ Significant adjusted mean allelic effects on total vBMD in subset analyses at 24 months: rs13312770: In Group_10_; B: 22.4 (95% CI, 3.4–41.4), *p* = 0.021.

^l^ Significant adjusted mean allelic effects on total CSA in subset analyses: At 12 months: rs7955866: In Group_10_: B: 11.4 (95% CI, 1.0–21.8), *p* = 0.032. In boys: B: 10.6 (95% CI, 0.2–20.9), *p* = 0.045. At 24 months: rs13312770: In Group_30_; B: 6.0 (95% CI, 0.0–12.0), *p* = 0.049.

^m^ Significant adjusted mean allelic effects on PMI in subset analyses: At 12 months: rs7955866: In Group_10_: B: 487 (95% CI, 18–956), *p* = 0.042. At 24 months: rs13312770: In Group_30_; B: 320 (95% CI, 15–625), *p* = 0.040.

Number of subjects in analyses (all/boys/girls): At 12 months: Rs7955866 and rs11063112: Phosphate (467/216/251), other variables (469/214/255). Rs11312770: Phosphate (435/204/231), other variables (442/200/242).

At 24 months: Rs7955866 and rs11063112: Parathyroid hormone (545/257/288), other variables (588/274/314).

Rs11312770: Parathyroid hormone (513/238/278), other variables (550/254/296).

iFGF23, intact fibroblast growth factor 23; cFGF23, C-terminal fibroblast growth factor 23; PTH, parathyroid hormone; BMC, bone mineral content; vBMD, volumetric bone mineral density; CSA, cross-sectional area of the bone.

#### 3.3.1 Biochemical parameters

Concentrations of 25-OHD modified iFGF23 and PTH in linear regression but did not affect other studied parameters. The genotype of the studied three SNPs did not affect 25-OHD, Ca ion, or iron concentrations (data not shown).

##### 3.3.1.1 Associations with *SNP1*

The *SNP1* genotype was associated with unadjusted cFGF23 in all participants and at both time points. *SNP1* minor allele homozygotes (AA) had lower cFGF23 concentrations than major allele homozygotes (GG) at 12 and 24 months (*p* = 0.005 for both), and a similar association was observed for the corresponding haplotypes (GAT vs. AAT), significant at 12 months (*p* = 0.034) (Table 4). Genotype effects of *SNP1* on the concentration of unadjusted cFGF23 were significant, also separately in boys and in Group_30_ at 12 and 24 months (*p* < 0.044 for all comparisons) (Table 4).

When examined by multivariate linear regression, using sex, iron, ionized calcium, and 25-OHD, as covariates, the adjusted mean allelic effects of *SNP1* genotype in all participants were significant for iFGF23 at 24 months (B = 5.56; *p* = 0.045), and separately in Group_10_ (B = 12.95; *p* = 0.021) and in girls (B = 11.72; *p* = 0.010), with a shift from major (G) to minor (A) alleles, thus increasing iFGF23 (Table 6). As the residuals of cFGF23 did not show normal distribution when analyzed by the *SNP1* genotype, secondary analyses were performed using logarithmic conversions of cFGF23 concentrations. The adjusted mean allelic effects for cFGF23 were significant in all participants at 24 months (*p* = 0.024), with a decrease in cFGF23 with the shift from major to minor *SNP1* alleles.

In Group_10_, minor allele homozygotes of *SNP1* had highest unadjusted phosphate concentration (*p* = 0.012) at 12 months and lowest unadjusted PTH at both time points (*p* < 0.004 for both). Unadjusted PTH was also lowest in boys with minor allele homozygotes at 24 months (*p* = 0.027) (Table 4). The adjusted mean allelic effects on phosphate were significant at 12 months in all participants (B = 0.04; *p* = 0.027) and in Group_10_ (B = 0.06; *p* = 0.011). Minor alleles were associated with increasing phosphate concentrations (Table 6).

##### 3.3.1.2 Associations with *SNP2*

Adjusted mean allelic effects of *SNP2* on cFGF23 at 12 months were significant in all participants (B = 0.23; *p* = 0.030) and separately in girls (B = 0.30; *p* = 0.036), but not in boys. Association with phosphate at 12 months was also evident in all participants (B = 0.03; *p* = 0.017) and separately in girls (B = 0.03; *p* = 0.043), as well as in Group_10_ (B = 0.05; *p* = 0.001). In Group_10_, unadjusted phosphate at 12 months was also related to the genotype of *SNP2* (*p* = 0.003), with heterozygotes (TA) having higher phosphate concentrations than major homozygotes (TT) (Table 6).

##### 3.3.1.3 Associations with *SNP3*

In multivariate linear regression, the *SNP3* genotype modified iFGF23 (B = −4.61, *p* = 0.006) in girls at 12 months, with a decrease in iFGF23 observed with the shift from major (T) to minor (C) alleles (Table 6).

##### 3.3.1.4 Associations with haplotypes

Unadjusted haplotype analyses mirrored the differences found in SNP analyses, with differences in PTH concentrations observed in Group_10_ (*p* = 0.008) and those in phosphate observed in boys (*p* = 0.034) at 12 months (Table 4).

#### 3.3.2 Bone parameters

The genotype of *SNP1* and *SNP2* did not affect unadjusted means or have significant adjusted mean allelic effects on pQCT-derived bone parameters when analyzed in all participants at 12 or 24 months.

##### 3.3.2.1 Associations with *SNP1*

In Group_10_, the *SNP1* genotype modified total BMC (*p* = 0.006), total CSA (*p* = 0.012), and PMI (*p* = 0.006) at 12 months, with minor homozygotes showing highest unadjusted means (Table 5). In multivariate linear regression, the *SNP1* genotype correspondingly modified total CSA and PMI in Group_10_ (B = 11.4; *p* = 0.032 and B = 487; *p* = 0.042) and total CSA in boys (B = 10.6; *p* = 0.045) at 12 months, with an increase in means related to the shift from major to minor alleles (Table 6). In girls, minor allele homozygosity of *SNP1* was also related to highest total vBMD (*p* < 0.001) at 24 months (Table 5).

##### 3.3.2.2 Associations with *SNP2*

In subset analyses, the *SNP2* genotype was associated with total BMC; minor allele homozygotes had lower means than major homozygotes in Group_10_ (*p* = 0.031) at 12 months, and in boys at both 12 and 24 months (*p* < 0.006 for both) (Table 5). In linear regression, a shift from major to minor alleles of *SNP2* correspondingly decreased the total BMC in boys at 24 months (B-1.9; *p* = 0.008) (Table 6)*.* At 24 months, major allele homozygosity of *SNP2* was also related to highest unadjusted total vBMD in boys (*p* = 0.032) (Table 5).

##### 3.3.2.3 Associations with *SNP3*

When studied in all participants, the genotype of *SNP3* was significantly associated with total BMC (*p* = 0.005), total CSA (*p* = 0.037), and PMI (*p* = 0.036) at 24 months, with highest unadjusted means observed in heterozygotes (TC). In subset analyses, these associations were significant for total BMC in boys and in Group_10_ (*p* = 0.037 and *p* = 0.024, respectively) and for total CSA and PMI in Group_30_ (*p* = 0.005 for both) (Table 5). In all participants, significant adjusted mean allelic effects on bone parameters were similarly observed for *SNP3*, with a shift from major to minor alleles increasing the total BMC at 24 months (B = 1.4; *p* = 0.028). In Group_10_, *SNP3* minor allele homozygotes had the highest unadjusted total vBMD (*p* = 0.045) (Table 5), and a shift from major to minor alleles of *SNP3* increased total vBMD (B = 22.4; *p* = 0.021) at 24 months (Table 6). In Group_30_, minor allele carriers also had greater total CSA and PMI (B = 6.0; *p* = 0.049 and B = 320; *p* = 0.040, respectively) at 24 months (Table 6).

##### 3.3.2.4 Associations with haplotype

In haplotype analyses, we did not observe effects on unadjusted bone parameters.

### 3.4 Genotype and temporal change in biochemical and bone parameters

#### 3.4.1 Temporal change in biochemical parameters

**FIGURE 4:**
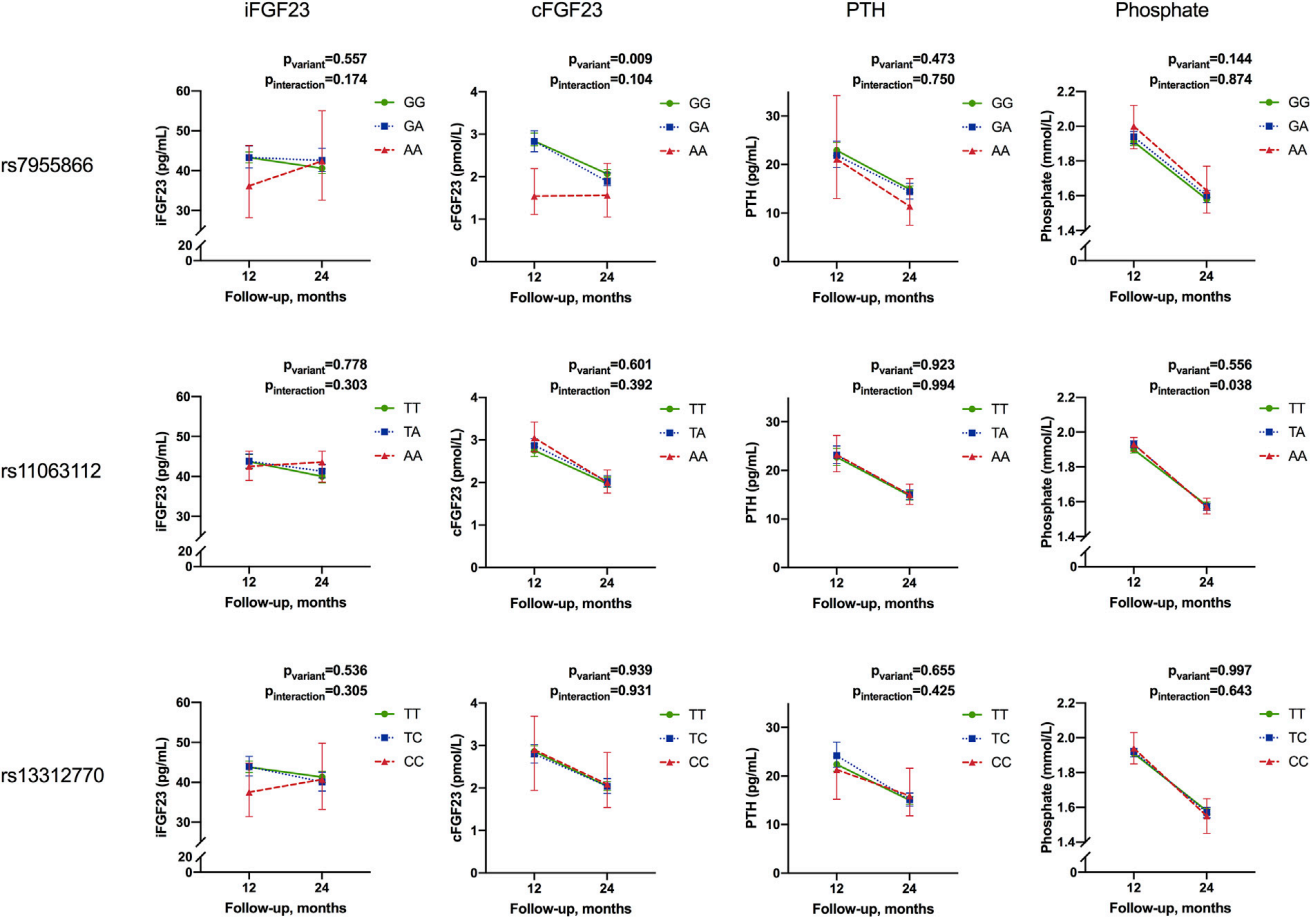
Temporal change in mean concentrations of intact FGF23, C-terminal FGF23, parathyroid hormone, and phosphate from 12 to 24 months by the genotype of studied SNPs. Results for linear mixed-model analyses of repeated measurements for differences in adjusted means (p_variant_) and differences in temporal change between variants (i.e., interaction of the variant and temporal change (P_interaction_). Results are given as adjusted mean concentrations and 95% confidence intervals (95% CI). Means are adjusted for season, sex, and interaction of time and season; 25-hydroxyvitamin D and ionized calcium for iFGF23 and PTH; iron for iFGF23; cFGF23 and phosphate and length-adjusted weight standard deviation score for phosphate (iFGF23, intact fibroblast growth factor 23; cFGF23, C-terminal fibroblast growth factor 23; PTH, parathyroid hormone).

Adjusted mean concentrations of biochemical parameters and results for linear mixed-model analyses are presented in Table 7 and Figure 4, respectively

**TABLE 7:** Temporal change of biochemical parameters during follow-up by genotype.

	12 months			24 months			P_variant_	P_interaction_
**rs7955866**	GG	**GA**	**AA**	**GG**	**GA**	**AA**		
**iFGF23 (pg/mL)^a^** ^**,**^ **^b^**	43.3 (42.0; 44.7)	43.3 (40.7; 46.2)	36.2 (28.2; 46.3)	40.6 (39.3; 41.9)	42.6 (39.8; 45.6)	42.4 (32.6; 55.0)	0.557^**c**^	0.174
**cFGF23 (pmol/L)^a^** ^**,**^ **^d^**	2.84 (2.73; 3.03)	2.83 (2.59; 3.08)	1.54 (1.11; 2.19)	2.07 (1.97; 2.17)	1.89 (1.79; 2.09)	1.56 (1.05; 2.31)	**0.009** ^**e**^	0.104
**PTH (pg/mL)^a^** ^**,**^ **^f^**	22.9 (21.8; 24.5)	21.9 (19.4; 24.8)	21.1 (13.0; 34.2)	14.9 (14.2; 15.6)	14.4 (12.9; 16.1)	11.4 (7.5; 17.1)	0.473	0.750
**Phosphate (mmol/L)^g^**	1.91 (1.89; 1.92)	1.94 (1.90; 1.97)	2.00 (1.87; 2.12)	1.58 (1.56; 1.59)	1.60 (1.56; 1.63)	1.63 (1.50; 1.77)	0.144	0.874
**rs11063112**	**TT**	**TA**	**AA**	**TT**	**TA**	**AA**		
**iFGF23 (pg/mL) ^a^** ^**,**^ **^b^**	43.7 (42.0; 45.5)	43.8 (42.1; 45.6)	42.5 (39.0; 46.3)	40.0 (38.4; 41.8)	41.3 (39.6; 43.0)	43.6 (38.6; 46.3)	0.778	0.303
**cFGF23 (pmol/L)^a^** ^**,**^ **^d^**	2.75 (2.61; 2.91)	2.87 (2.72; 3.03)	3.05 (2.72; 3.42)	1.97 (1.89; 2.13)	2.03 (1.90; 2.16)	2.00 (1.75; 2.29)	0.601	0.392
**PTH (pg/mL) ^a^** ^**,**^ **^f^**	22.7 (21.0; 24.5)	23.1 (21.4; 25.0)	23.2 (19.7; 27.2)	14.8 (13.9; 15.8)	15.0 (14.0; 16.0)	15.0 (13.0; 17.2)	0.923	0.994
**Phosphate (mmol/L)^g^**	1.90 (1.88; 1.92)	1.93 (1.91; 1.95)	1.93 (1.89; 1.97)	1.58 (1.56; 1.60)	1.57 (1.55; 1.59)	1.57 (1.53; 1.62)	0.556	**0.038**
**rs13312770**	**TT**	**TC**	**CC**	**TT**	**TC**	**CC**		
**iFGF23 (pg/mL)^a^** ^**,**^ **^b^**	43.8 (42.4; 45.3)	43.9 (41.6; 46.5)	37.5 (31.4; 44.8)	41.3 (39.9; 42.8)	40.1 (37.8; 42.5)	40.7 (33.2; 49.8)	0.526	0.305
**cFGF23 (pmol/L)^a^** ^**,**^ **^d^**	2.86 (2.73; 2.99)	2.80 (2.59; 3.02)	2.90 (2.28; 3.69)	2.04 (1.94; 2.15)	2.04 (1.87; 2.22)	2.09 (1.54; 2.84)	0.939	0.931
**PTH (pg/mL)^a^** ^**,**^ **^f^**	22.4 (21.0; 23.9)	24.2 (21.8; 27.0)	21.3 (15.2; 20.9)	15.0 (14.2; 15.8)	15.1 (13.8; 16.5)	15.9 (11.8; 21.6)	0.655	0.425
**Phosphate (mmol/L)^g^**	1.91 (1.90; 1.93)	1.92 (1.89; 1.94)	1.94 (1.85; 2.03)	1.58 (1.56; 1.59)	1.57 (1.54; 1.60)	1.55 (1.45; 1.65)	0.997	0.643 **^h^**

Results for linear mixed-model analyses of repeated measurements for differences in adjusted means of studied biochemical parameters (p_variant_) and differences in temporal change between variants (i.e., interaction of variant and temporal change (P_interaction_)). Results are expressed as adjusted mean concentrations and 95% confidence intervals (95% CI). Significant p-values are highlighted in bold.

^a^ Back-transformed from logarithmic values.

^b^ Covariates: Season at follow-up (<0.014), iron (<0.001), sex (<0.001), and interaction of time and season (<0.001) for all SNPs; 25-hydroxyvitamin D (<0.044) for rs7955866 and rs11063112 and ionized calcium (0.040) for rs7955866.

^c^ In girls: P_variant_ = 0.041. Significant differences in multiple comparisons (Bonferroni) GG vs. GA (*p* = 0.046, mean difference: −0.084).

^d^ Covariates: Season at follow-up (<0.004), iron (<0.001) for all SNPs, and interaction of time and season (0.023) for rs7955866.

^e^ Significant differences in multiple comparisons (Bonferroni) AA vs. GA and GG (Bonferroni *p* = 0.037 and 0.012 respectively, mean difference: −0.760 and −0.727, respectively). In boys: P_variant_ = 0.027 (multiple comparisons non-significant). In girls: P_variant_ = 0.016. Significant differences (Bonferroni) AA vs. GA and GG *p* , 0.012 and 0.016, respectively, mean difference: −0.399 and −0.447, respectively).

^f^ Covariates: 25-hydroxyvitamin D (<0.001), ionized calcium (<0.001), season at follow-up (0.009), and sex (<0.004) for all SNPs; interaction of time and season (<0.023) for rs7955866 and 11063112.

^g^ Covariates: iron (<0.001) for all SNPs, season at follow-up (0.014), and length-adjusted weight SDS (0.017) for rs13312770.

^h^ In boys: P_interaction_ = 0.022.

Number of subjects in analyses (rs7955866/rs11063112/rs13312770): 579/576/544.

iFGF23 = intact FGF23; cFGF23 = C-terminal FGF23; PTH = parathyroid hormone.

Concentrations of iFGF23, cFGF23, PTH, and phosphate decreased from 12 to 24 months in the study participants. Concentrations varied by season; iFGF23, cFGF23, and phosphate were highest in winter (*p* = 0.024, 0.036, and 0.037, respectively), while 25-OHD was lowest and PTH highest in spring (*p* = 0.010 and 0.002, respectively) (data not shown). The studied SNPs did not affect concentrations or temporal change of 25-OHD, Ca ion, or iron (data not shown).

In the whole cohort, genotype effects in linear mixed-model analyses were significant only for *SNP1* and cFGF23 concentrations (*p* = 0.009), with lowest concentrations linked to minor allele homozygosity. However, although not relating with adjusted cross-sectional concentrations, the *SNP2* genotype was found to be associated with a change in phosphate from 12 to 24 months, with a greatest decrease observed in minor allele homozygotes (p_interaction_ = 0.038) (Table 7; Figure 4).

In subset analyses, the *SNP1* genotype was also associated with iFGF23 concentrations, but not a temporal change, in girls (p_variant_ = 0.041), with heterozygotes showing the greatest adjusted mean concentrations. In boys, minor allele homozygosity of *SNP3* was related to greatest decrease in phosphate concentrations (p_interaction_ = 0.022) (Table 7).

The haplotype did not modify the temporal changes in iFGF23, cFGF23, PTH, or phosphate.

#### 3.4.2 Temporal change in bone parameters

**FIGURE 5:**
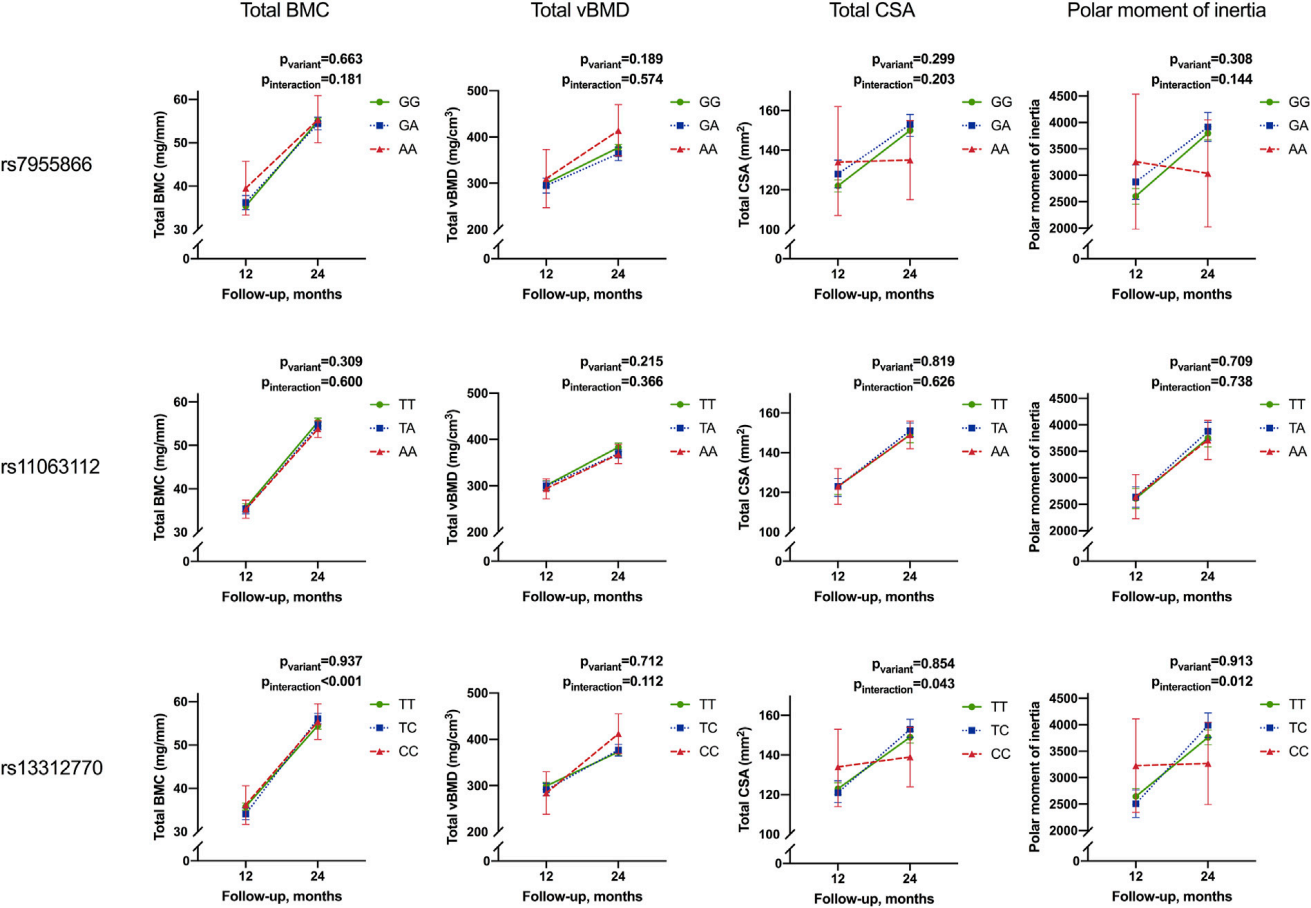
Temporal change in mean Total BMC, total vBMD, total CSA, and PMI from 12 to 24 months by the genotype of studied SNPs. Results for linear mixed-model analyses of repeated measurements for differences in adjusted means (p_variant_) and differences in the temporal change between variants (i.e., interaction of the variant and temporal change (P_interaction_). Results are given as adjusted means and 95% confidence intervals (95% CI). Means are adjusted for season at follow-up and length-adjusted weight standard deviation score for all bone parameters and sex for total BMC, total CSA, and PMI (BMC, bone mineral content; vBMD, volumetric bone mineral density; CSA, cross-sectional area of the bone).

Adjusted mean concentrations of bone parameters and results for linear mixed-model analyses are presented in Table 8 and Figure 5. The means of bone parameters increased with age during follow-up. Total BMC, total CSA, and PMI were lowest in spring (*p* < 0.009), while total vBMD was the lowest in winter (*p* = 0.049) (data not shown).

**TABLE 8:** Temporal change in bone parameters during follow-up by the genotype.

	12 months			24 months			P_variant_	P_interaction_
**rs7955866**	GG	**GA**	**AA**	**GG**	**GA**	**AA**		
**Total BMC (mg/mm)^a^**	35.4 (34.7; 36.1)	36.2 (34.6; 37.8)	39.5 (33.3; 45.7)	55.0 (54.4; 55.7)	54.4 (53.0; 55.9)	55.3 (50.0; 60.6)	0.663 **^b^**	0.181
**Total vBMD (mg/cm** ^**3**^ **)^c^**	300 (292; 307)	295 (279; 311)	310 (247; 373)	377 (371; 384)	364 (349; 378)	414 (359; 470)	0.189	0.574
**Total CSA (mm** ^**2**^ **)^a^**	122 (119; 125)	128 (121; 135)	134 (107; 162)	150 (147; 152)	153 (147; 158)	135 (115; 155)	0.299 **^d^**	0.203 **^d^**
**Polar moment of inertia^a^**	2,603 (2,454; 2,751)	2,873 (2,547; 3,198)	3,257 (1,980; 4,536)	3,796 (3,671; 3,921)	3,916 (3,644; 4,189)	3,036 (2,024; 4,049)	0.308 **^e^**	0.144 **^e^**
**rs11063112**	**TT**	**TA**	**AA**	**TT**	**TA**	**AA**		
**Total BMC (mg/mm)^a^**	35.7 (34.8; 36.6)	35.2 (34.2; 36.1)	35.3 (33.2; 37.4)	55.4 (54.5; 56.3)	54.5 (53.6; 55.4)	53.8 (51.8; 55.7)	0.309 **^f^**	0.600
**Total vBMD (mg/cm** ^**3**^ **)^c^**	301 (291; 311)	300 (290; 310)	294 (272; 315)	383 (374; 392)	369 (360; 378)	368 (348; 389)	0.215 **^g^**	0.366
**Total CSA (mm** ^**2**^ **)^a^**	123 (119; 127)	123 (118; 127)	123 (114; 132)	149 (145; 152)	151 (148; 155)	149 (142; 156)	0.819	0.626
**Polar moment of inertia^a^**	2,610 (2,420; 2,801)	2,636 (2,444; 2,829)	2,645 (2,228; 3,063)	3,748 (3,583; 3,913)	3,878 (3,709; 4,048)	3,715 (3,344; 4,086)	0.709	0.738
**rs13312770**	**TT**	**TC**	**CC**	**TT**	**TC**	**CC**		
**Total BMC (mg/mm)^a^**	35.8 (35.0; 36.6)	34.1 (32.8; 35.5)	36.2 (31.7; 40.6)	54.5 (53.7; 55.2)	56.1 (54.9; 57.3)	55.4 (51.3; 59.5)	0.937	**<0.001** ^**h**^
**Total vBMD (mg/cm** ^**3**^ **)^c^**	299 (292; 307)	291 (278; 305)	284 (238; 330)	373 (365; 380)	377 (364; 389)	412 (369; 455)	0.712	0.112
**Total CSA (mm** ^**2**^ **)^a^**	123 (120; 126)	121 (116; 127)	134 (114; 153)	149 (146; 152)	153 (149; 158)	139 (124; 154)	0.854	**0.043** ^**i**^
**Polar moment of inertia^a^**	2,644 (2,493; 2,794)	2,507 (2,246; 2,768)	3,226 (2,342; 4,111)	3,762 (3,622; 3,901)	3,993 (3,762; 4,223)	3,268 (2,493; 4,042)	0.913	**0.012** ^**j**^

Results for linear mixed-model analyses of repeated measurements for differences in adjusted means of studied bone parameters (p_variant_) and differences in temporal change between variants (i.e., interaction of variant and temporal change (P_interaction_)). Results are expressed as adjusted means and 95% confidence intervals (95% CI). Significant p-values are highlighted in bold.

^a^ Covariates: sex (<0.005), length-adjusted weight SDS (<0.001), season at follow-up (<0.032).

^b^ In Group_10_: P_variant_ = 0.046, significant differences (Bonferroni) AA vs. GA and GG (*p* = 0.041 for both, mean difference: 12.1 and 11.9, respectively).

^c^ Covariates: season at follow-up (<0.020) for rs7955866 and rs11063112, length-adjusted weight SDS (0.028) for rs13312770.

^d^ In Group_10_: P_variant_ = 0.021 (multiple comparisons, non-significant), p_interaction_ = 0.037.

^e^ In Group_10_: *p* = 0.024 (multiple comparisons, non-significant), p_interaction_ = 0.020.

^f^ In boys: P_varian**t**_ = 0.018 (multiple comparisons, non-significant).

^g^ In boys: P_varian**t**_ = 0.015 (significant differences (Bonferroni) TT vs. TA (*p* = 0.014, mean difference: 21.0).

^h^ In Group_10_: P_interaction_ = 0.002. In boys: P_interaction_ = 0.012. In girls: P_interaction_ = 0.029.

^i^ In Group_30_: P_interaction_ = 0.019.

j In Group_30_: P_interaction_ = 0.005. In boys: P_interaction_ = 0.034.

Number of subjects in analyses (rs7955866/rs11063112/rs13312770): 592/588/555.

BMC, bone mineral content; vBMD, volumetric bone mineral density; CSA, cross-sectional area of the bone.

The genotype of *SNP1* or *SNP2* did not modify the adjusted means or the increase in bone parameters in all participants. When stratified by the intervention group, the effects of *SNP1* on total BMC, total CSA, and PMI were significant in Group_10_ (p_variant_ = 0.046, 0.021, and 0.024, respectively). Minor allele homozygotes had the greatest adjusted means of total BMC, total CSA, and PMI and the smallest increase in total CSA and PMI (p_interaction_ = 0.037 and 0.020, respectively) during follow-up (Table 8).

In boys, *SNP2* major allele homozygotes had the greatest mean total BMC and vBMD (p_variant_ = 0.018 and 0.015, respectively) (Table 8).

*SNP3* significantly modified the temporal change in total BMC, total CSA, and PMI in all participants (p_interaction_ <0.001, 0.043, and 0.012, respectively). Minor allele homozygotes had the smallest increase in total CSA and PMI, while heterozygotes showed the greatest increase in total BMC (Table 8; Figure 5).

The observed effect of the *SNP3* genotype on increase in total BMC was also significant in Group_10,_ and in girls and boys separately (p_interaction_ = 0.002, 0.012, and 0.029, respectively). The effect on total CSA and PMI was also significant separately in Group_30_ (p_interaction_ = 0.019 and 0.005, respectively) and in boys for the PMI (p_interaction_ = 0.034) (Table 8).

The haplotype effects on the temporal change in bone parameters were not significant.

## 4 Discussion

This study finds that, in healthy young children, common *FGF23* SNPs rs7955866 (*SNP1*), rs11063112 (*SNP2*), and rs13312770 (*SNP3*) are associated with circulating FGF23 and phosphate concentrations and with pQCT-derived bone strength parameters, as well as with change in bone strength from 12 to 24 months. In all participants, *SNP1* minor allele homozygosity was related to lower cFGF23 concentrations at both 12 and 24 months of age. *SNP3* minor allele carriers had greater total BMC and showed greater increase in total BMC, but smaller increase in total CSA and PMI, from 12 to 24 months. In addition, the *SNP2* genotype was associated with change in phosphate from 12 to 24 months, with the greatest decrease in concentrations observed in minor allele carriers. Further genotype associations were observed only in participants receiving standard vitamin D supplementation, although vitamin D intervention did not affect the studied bone strength or biochemical parameters, apart from 25-OHD and PTH. Concentrations of 25-OHD were not modified by the studied *FGF23* SNP genotypes.

Previous GWAS studies in adults have found SNPs near *FGF23*, *SLC34A1*, and *CASR* to relate to differences in phosphate concentrations (Kestenbaum et al., 2010). While direct associations of *FGF23 SNP2* or *SNP3* with phosphate metabolism have not previously been described, minor allele homozygosity of *SNP1* is related to lower phosphate concentrations in adult nephrolithiasis patients and a smaller cohort of school-aged children (Rendina et al., 2012; Pekkinen et al., 2015). SNPs linked to genes involved in vitamin D metabolism; cleavage and inactivation of FGF23 (*PCSK9*); and cell proliferation, inflammation, and apoptosis (*HLA-DQA1*, *HLA-DQB1*, and *TGFB2,* respectively) have, in turn, are associated with circulating FGF23 concentrations (White et al., 2001; Benet-Pagès et al., 2004; Robinson-Cohen et al., 2018; Chuang et al., 2020). Apart from confirming the associations of *FGF23* genetic variation with phosphate metabolism in the studied age group, our study is, to our knowledge, the first to show a direct association between variation in the *FGF23* gene and FGF23 concentrations.

In all participants, minor alleles were related to lower cFGF23 throughout follow-up (S*NP1*) and positive allelic effects on phosphate at 12 months of age (*SNP1-2*), but the greatest decrease in phosphate was observed from 12 to 24 months (*SNP2* in all*, SNP3* in boys). In analyses by sex, *FGF23* minor allele carriers showed lowest iFGF23 in boys (*SNP2*) and negative allelic effects in girls (*SNP3*). The *FGF23* genotype also notably affected bone strength and age-dependent change thereof. Minor alleles related to greater increase of total BMC, but smaller increase of total CSA and PMI, compared with major allele homozygotes, with heterozygotes having highest total BMC, CSA, and PMI at 24 months (*SNP3*). In contrast to *SNP3*, in boys, minor allele homozygosity of *SNP2* was observed to be associated with lower total BMC at both 12 and 24 months, and lower total vBMD at 24 months.

The genotype-related differences in phosphate concentrations at 12 months differ from those previously reported in adult nephrolithiasis patients and a smaller cohort of school-aged children (Rendina et al., 2012; Pekkinen et al., 2015), with minor allele homozygosity relating to higher, instead of lower, phosphate concentration. We have previously found phosphate concentrations to markedly decrease from 12 to 24 months of age (Koljonen et al., 2021). In light of the age-related decrease in phosphate, which is greater in *FGF23* minor allele carriers (*SNP2-3*), the different direction of genotype associations observed at 12 vs. 24 months of age, and the simultaneously decreasing FGF23 and PTH concentrations, the differing findings could be explained by age-specific regulation.

Differences in the direction of genotype associations at 12 and 24 months are similarly observed for several bone parameters. Significant differences in temporal change, but not in cross-sectional adjusted means, as observed, for example., for *SNP3* and total BMC, CSA, and PMI, are indicative of differences in genotype effects at the two studied time points. Growth velocity in infancy is greater in boys and decreases after 12 months of age (Kiviranta et al., 2016), while physical activity increases, including standing and onset of walking, that previously have been shown to be associated with bone strength in early childhood (Ireland et al., 2014). Observed differences between the sexes possibly reflect differences in growth velocity, as well as differences in FGF23 regulation previously observed between girls and boys in the studied age group (Holmlund-Suila et al., 2017; Enlund-Cerullo et al., 2020). We hypothesize that the differences in genotype effects, observed both for studied biochemical and bone parameters, potentially relate to age-specific differences in regulation, as well as to differences in growth phase and physical activity at 12 and 24 months.

The observed associations of the *FGF23* genotype and cFGF23 concentrations in all participants could potentially indicate reduced expression of iFGF23, with correspondingly higher phosphate concentration as observed at 12 months, in minor allele carriers (*SNP1*), potentially related to altered binding to the FGF receptor and Klotho complex (Yamazaki et al., 2008; Rendina et al., 2012). The reduced expression of iFGF23 could be balanced by a decrease in cleavage, resulting in non-significant differences in iFGF23 by the genotype, but subsequently observed as a decrease in cFGF23 in minor allele carriers. Findings of minor allele genotype associations with low iFGF23 at 12 months observed in analyses by sex potentially support this hypothesis.

In limited adult studies, iFGF23 concentrations have been found to be negatively associated with BMD (Bouksila et al., 2019; Wang et al., 2020). Apart from its effects on phosphate metabolism, *in vitro* studies have suggested FGF23 expression to independently regulate bone formation (Wang et al., 2008). Inverse association of iFGF23 with BMD parameters has also recently been observed in a Mendelian randomization study exploring causal effects of FGF23 (Yokomoto-Umakoshi et al., 2021). As our study setting does not allow us to establish causality, the mechanisms underlying the observed differences between *FGF23* genotype and bone strength and temporal change, thereof, require further studies. Based on earlier reports (Bouksila et al., 2019; Wang et al., 2020; Yokomoto-Umakoshi et al., 2021), the observed genotype effects on bone parameters could potentially correspond with effects on iFGF23 expression and concentrations. Our previous study in a smaller cohort of school-aged children observed associations of FGF23 diplotype and total hip BMD Z-scores (Pekkinen et al., 2015). The present study finds variation in *FGF23* in infants to be primarily associated with differences in total BMC, total CSA, and PMI, i.e., measures of bone size, mass, and resistance to torsion, rather than with volumetric bone mineral density (total vBMD), for which associations are observed only in Group_10_ (*SNP3*).

Considering the tight interplay between vitamin D, phosphate, and bone metabolism, our study uniquely explores the effects of genetic variation in *FGF23* in a vitamin D intervention setting. In our largely vitamin D sufficient study population, vitamin D intervention affected 25-OHD and PTH levels, as previously reported, but did not directly modify other biochemical or bone parameters (Rosendahl et al., 2018; Valkama et al., 2020). Some of our findings were, however, specific for participants receiving standard vitamin D supplementation (Group_10_). In contrast to our earlier study in school-aged children, *FGF23* genotype did not modify 25-OHD, but in Group_10_, minor alleles associated with lower PTH (*SNP1*), a finding in line with our previous report (Pekkinen et al., 2015). As vitamin D intervention had a significant effect on PTH and 25-OHD concentration, it is plausible that the impact of higher vitamin D supplementation in Group_30_ did not allow detection of possible smaller effects of the *SNP1* genotype on PTH and 25-OHD concentrations. We have previously found 25-OHD to modify iFGF23 in girls, while seasonal differences, possibly reflecting changes in vitamin D status, are associated with iFGF23 concentrations in boys (Enlund-Cerullo et al., 2020). In the present study, 25-OHD modified adjusted iFGF23 concentrations by the *SNP1* genotype. Indirect effects of 25-OHD are possibly also reflected in the seasonal differences observed in bone parameters and iFGF23 and cFGF23 concentrations.

It is interesting to note that we observe positive effects on bone strength parameters for *SNP1* only in participants receiving standard, but not high-dose, vitamin D supplementation. In Group_10_, minor allele homozygosity was related to higher total BMC, CSA, and PMI at 12 months and higher total BMC but lower CSA at 24 months, and smaller increase of total CSA and PMI from 12 to 24 months (*SNP1*), largely mirroring the findings in all participants (*SNP3*). As effects of *SNP1* include potential differences in binding of FGF23 to FGF receptors and Klotho (Yamazaki et al., 2008; Rendina et al., 2012), it can be speculated that interaction with vitamin D and its metabolites is modified in minor allele carriers, possibly depending on 25-OHD and vitamin D supplementation dose. Further studies are needed to explore the interplay of vitamin D, FGF23, and genetic variation in *FGF23* with bone strength and modeling in young children.

When considering observed associations between singular SNPs and phenotype, it is to be noted that associations may, in part, be due to linkage disequilibrium between studied and other genetic variants. In our study, potential differences in expression and post-translational modification of FGF23 through altered binding to the FGF receptor and Klotho and consequent differences in signaling (*SNP1*) (Yamazaki et al., 2008; Rendina et al., 2012) and possible differences in susceptibility to O-glycosylation (*SNP2*) may directly contribute to the observed differences in biochemical and bone parameters. The associations of *SNP3* variation with bone parameters could possibly reflect minute effects on FGF23 function and PTH and phosphate concentrations, that were not statistically detectable.

The large cohort of healthy infants with uniform and extensive follow-up is a notable strength of this study, uniquely allowing us to examine genotype effects on temporal changes, in addition to cross-sectional associations, of the studied biochemical and bone parameters. Despite the cohort size, minor allele homozygotes of the studied SNPs, and consequently their haplotypes, were, however, rare, making detection of significant genotype effects challenging and arguably inconclusive, for example, for variants where heterozygotes show greatest means. It is possible that some genotype associations may have escaped detection, and larger studies are warranted to further examine the associations of *FGF23* variation and mineral and bone metabolism. Although the intervention setting allowed us to examine the potential impact of different vitamin D supplementation doses, direct supplementation effects on, for example, 25-OHD and PTH concentrations may have made detection of smaller, *FGF23* genotype-derived associations more challenging, especially in the higher-dose supplementation group. Analyses of 1,25-(OH)_2_D, urinary phosphate, creatinine, or calcium were unfortunately not conducted in this study.

Our study presents novel associations of FGF23 and phosphate concentrations and bone strength with common SNPs of *FGF23* in healthy infants. Our findings highlight the role of *FGF23* in bone modeling, in particular effects on bone mineral content, cross-sectional area, and PMI, in growing young children. As simple measurements of FGF23 concentrations are subject to many confounding factors, our findings can help understand the role of FGF23 in bone metabolism and its temporal changes during early childhood.

## Data Availability

Data cannot be shared publicly because the data consists of sensitive patient data. More specifically the data consists of individual clinical data and individual genotypes for young children. Data are available from the Helsinki University Hospital's Institutional Data Access/Ethics Committee for researchers who meet the criteria for access to confidential data. Data availability contacts: OM, outi.makitie@helsinki.fi.
