# Supplementary material for: Variation in the fibroblast growth factor 23 (FGF23) gene associates with serum FGF23 and bone strength in infants
Source: Front Genet. 2023 May 22;14:1192368. doi: 10.3389/fgene.2023.1192368 (PMC10240082; doi:10.3389/fgene.2023.1192368)
Supplement: Supplementary file 1 [file DataSheet1.docx]

Supplementary Material

Variation in the Fibroblast Growth Factor 23 (*FGF23)* gene associates with serum FGF23 and bone strength in infants

**Maria Enlund-Cerullo, MD, MSc *, Elisa Holmlund-Suila, MD, PhD, Saara Valkama, MD, Helena Hauta-alus, MSc, PhD, Jenni Rosendahl, MD, PhD, Sture Andersson, MD, PhD, Minna Pekkinen MSc, PhD, Outi Mäkitie, MD, PhD.**

*** Correspondence:** Corresponding Author: [maria.enlund@helsinki.fi](mailto:maria.enlund@helsinki.fi)


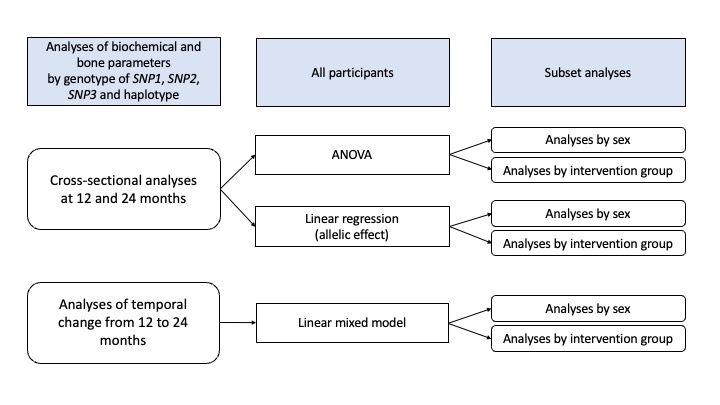


**Supplementary Figure 1.** **Performed analyses of biochemical and bone parameters by genotype of *FGF23* variants rs7955866 (*SNP1*), rs11063112 (*SNP2*) and rs13312770 (*SNP3*).**

Analyses of biochemical parameters (Intact FGF23, C-terminal FGF23, phosphate and parathyroid hormone) and peripheral quantitative computed tomography (pQCT) -derived bone parameters (Total bone mineral content, cross sectional area, volumetric bone mineral density and polar moment of inertia) were primarily performed in all participants and secondly by sex and intervention group. Cross-sectional analyses included analysis of covariance (ANOVA) and linear regression at 12 and 24 months. Temporal change from 12 to 24 months of age was examined by linear mixed model. Adjusted analyses (linear regression and linear mixed model) were perfomed using previously indicated modifying factors, significant covariates were included in each analysis.
